# Compensation effects between the apparent activation energy and pre-exponential factor in simple models of electrocatalytic hydrogen evolution

**DOI:** 10.1039/d5fd00163c

**Published:** 2026-01-15

**Authors:** Jan Fingerhut, Rifael Z. Snitkoff-Sol, Maximilian Albers, Rafaël E. Vos, Onno van der Heijden, Marc T. M. Koper

**Affiliations:** a Leiden Institute of Chemistry, Leiden University Einsteinweg 55 2333 CC Leiden The Netherlands m.koper@chem.leidenuniv.nl; b Department of Chemical Engineering and Chemistry, Technical University of Eindhoven 5600 MB Eindhoven The Netherlands

## Abstract

Understanding how activation energies and entropies vary with electrode potential is central to interpreting electrocatalytic kinetics, yet temperature-dependent analyses often yield apparent “compensation effects” whose physical origin remains debated. Here, we model the hydrogen evolution reaction (HER) through both Volmer–Heyrovsky and Volmer–Tafel mechanisms to determine under which conditions compensation effects emerge—even when the entropic contribution to the activation barriers of the individual reaction steps is, by assumption, potential-independent. By simulating steady state currents across realistic potential and temperature ranges and extracting apparent Arrhenius parameters, we identify two general origins of compensation-like behavior: (i) shifts in the effective rate law arising from changes in coverage or in the rate-determining step, and (ii) the presence of multiple active sites with different equilibrium potentials of reaction steps. Both phenomena generate non-constant Tafel slopes and curvature in Arrhenius plots, producing Constable-plot (log_10_ *A*_app_*vs. E*_app_) relations that mimic intrinsic entropy–enthalpy compensation. Our results demonstrate that compensation effects in electrocatalysis can arise purely from kinetic coupling and site heterogeneity rather than from fundamental thermodynamic scaling, underscoring the need for caution when interpreting temperature-dependent kinetic data. We propose that meaningful mechanistic insight requires measurements on well-defined single-crystal electrodes within potential regions where Tafel slopes remain constant.

## Abstract 2

The hydrogen evolution reaction (HER) is modeled through both Volmer–Heyrovsky and Volmer–Tafel mechanisms to determine under which conditions compensation effects emerge.

## Graphical abstract

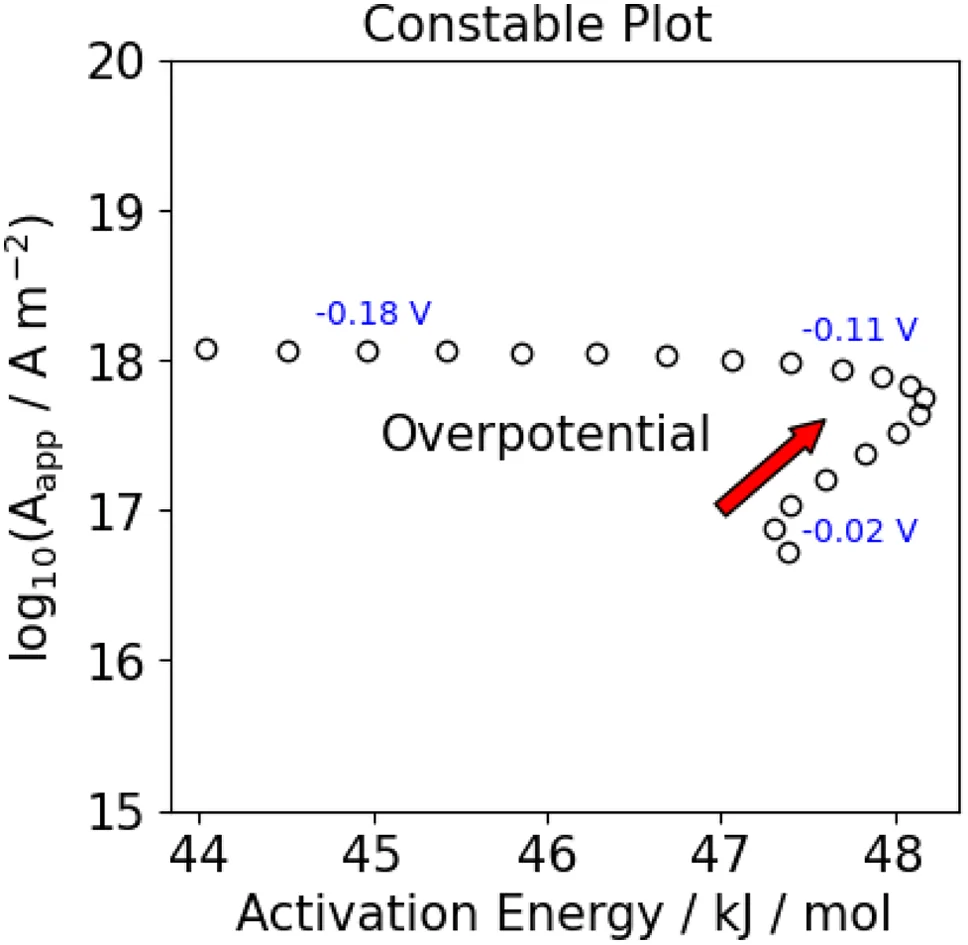

## Introduction

Elucidation of mechanisms and reaction parameters associated with (electro)catalytic reactions enables probing the underlying principles that govern the structure–reactivity relationship that is fundamental to (electro)catalysis. In electrocatalytic experiments, a key control parameter is the electrode potential, which is related to the thermodynamic driving force, *i.e.*, the Gibbs reaction free-energy by

1 Δ*G*_rxn_ = −*nF*(*E* − *E*^0^)

Applying an overpotential, *η* = (*E* − *E*^0^), induces a proportional change in the Gibbs free-energy difference between the initial and final states of the corresponding redox reaction (with *n* the number of electrons involved, and *F* the Faraday constant). The transformation from initial to final state requires overcoming an activation Gibbs free-energy barrier, *i.e.*, Δ*G*_act_, which can be reduced by increasing the thermodynamic driving force, *i.e.*, Δ*G*_rxn_. The cornerstone equation of electrode kinetics, *i.e.* the phenomenological Butler–Volmer (BV) equation, assumes a linear relationship between Δ*G*_rxn_ and Δ*G*_act_, equivalent to the Bronsted–Evans–Polanyi (BEP) relation:

2 Δ*G*_act_ = Δ*G*^*η*=0^_act_ ± *αF*(*E* − *E*^0^)

with *α* a Brønsted coefficient, known as the transfer coefficient or symmetry factor in electrode kinetics (and with the plus/negative sign corresponding to a reduction/oxidation reaction). The activation energy at zero thermodynamic driving force, Δ*G*^*η*=0^_act_, is the standard activation energy and governs the reaction rate, which is why quantitative determination of this parameter is essential. The reaction rate is related to the activation energy in an Arrhenius type law,3
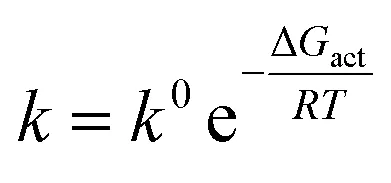
where *k*^0^ is the pre-exponential factor. Combining eqn (2) and (3) gives the BV equation.^1^

In most electrode kinetics studies, the focus is on deriving mechanistic information from the potential dependence of the reaction rate through the so-called Tafel analysis. Determining activation energies and their potential dependence, through Arrhenius analysis, is much less widespread. If we write Δ*G*_act_ in terms of enthalpy (Δ*H*_act_) and entropy (Δ*S*_act_) of activation

4 Δ*G*_act_ = Δ*H*_act_ − *T*Δ*S*_act_

constructing a plot of ln(*k*) *vs.* 1/*T*, *i.e.*, an Arrhenius plot,5
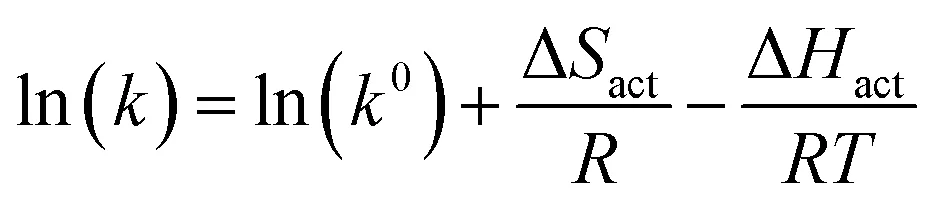


gives −Δ*H*_act_/*R* as the slope, and ln(*A*) = ln(*k*^0^) + Δ*S*_act_/*R* as the intercept. This simple manipulation shows that Arrhenius analysis in principle allows a separation between enthalpic and entropic contributions to the activation energy. Quite a few recent papers in electrocatalysis have suggested that this separation may hold crucial information on the nature of electrocatalytic charge transfer, and that manipulating activation entropy is an overlooked strategy towards improved electrocatalytic activity.^2–7^

Following eqn (2), Δ*H*_act_ and Δ*S*_act_ are expected to be electrode potential dependent as well. In the simplest, ideal, case the change in electrode potential only shifts the enthalpic contribution to the free energy and does not influence the entropic part, *i.e.*,

6 Δ*H*_act_ = Δ*H*^*η*=0^_act_ ± *αFη*

and Δ*S*_act_ ≠ *f*(*η*). In such a case, the intercepts of the Arrhenius plots (eqn (5)) constructed at different potentials should all fall on the same value, as they are insensitive to the potential.^8^ However, experimental reports of Arrhenius analysis show a strong potential dependence of the intercept.^3,4,9–13^ This would imply that the entropic part of the activation free energy is potential dependent as well. In such cases, plotting the experimentally determined activation energy (*E*^app^_act_ = Δ*H*_act_) *vs.* the intercept (ln(*A*)) often shows a linear dependence, which is a well-known phenomenon called the compensation effect.^14^

The compensation effect has been reported extensively in the broad field of heterogenous catalysis^15,16^ as well as in electrocatalysis.^3,4,10,17^ The potential dependence of the Arrhenius intercept (ln(*A*)) is closely linked to the anomalous temperature dependence of the Tafel slope, as shown by Conway’s studies^8^ of the hydrogen evolution reaction (HER) on various electrodes across wide temperature ranges.^18,19^ While Arrhenius plots are linear over narrow temperature intervals, significant curvature appears over broader ranges, correlating with changes in Tafel slopes. This behavior has been attributed by extensive theoretical work to temperature-dependent orientation or dynamics of solvent molecules, changes in the double-layer, proton tunneling and anion adsorption.^20–24^

While these effects are obviously of high interest and should be thoroughly investigated, drawing meaningful conclusions from experimental temperature dependent measurements is not trivial.^25^ Two relevant papers by Barrie showed that apparent compensation effects can arise from random errors in the data,^26^ as well as from systematic errors, such as a change in the coverage of a catalytic intermediate or a change in the rate-determining step (RDS).^14,27^ Moreover it was shown that apparent compensation effects arise when analyzing the total rate of the reaction on a catalyst that is composed of a distribution of active sites on which the reaction proceeds with different rates,^28,29^ as occurs on polycrystalline surfaces and catalytic nanoparticles.^30–32^ The electrochemical environment further complicates kinetics, as solvent, electrolyte, and impurities can adsorb on electrodes and alter reactions in a temperature-dependent manner. Furthermore, the Gileadi group has shown that an apparent compensation effect for HER on Hg disappears by correcting for double-layer potential drops^33–35^ and Zhang *et al.* showed that increasing electrolyte concentration can eliminate an apparent compensation effect for the CO reduction reaction.^36,37^ Additionally, the distribution of the potential in thick catalyst layers, such as in fuel cells or electrolyzers, is not accounted for in normal *iR* (current × resistance) compensation schemes.^38,39^

This paper was motivated by the question of what the applicability is of the compensation effect for studying fundamental properties of the electrocatalytic reaction rates, especially in combination with Tafel analysis. Specifically, a question that arises is whether in the absence of a linear region in a Tafel plot, *i.e.*, Tafel slope varying with potential (a situation we analyzed in detail in recent papers^13,40,41^), meaningful conclusions can be arrived at regarding the origin of the compensation. To this end, we modeled mathematically the simplest electrocatalytic reaction, *i.e.*, the hydrogen evolution reaction (HER), and studied the temperature dependence of its kinetics to uncover the mechanistic regimes where indeed an apparent compensation effect arises. From this, we show that a compensation effect can be seen if there is a change in balance between two steps as a function of potential or if multiple active sites are present on a catalyst. More importantly, these changes are not caused by a change in the entropic contribution to the individual reaction steps, showing that the apparent compensation effects are not related to an intrinsic enthalpy–entropy compensation.

## Model

To study the emergence of a compensation effect due to the kinetics of the HER, we model the kinetic current at steady state, assuming no potential dependence of the entropic part of the free-energy of activation. In different mechanistic regimes, we systematically investigate the occurrence of a compensation effect by varying the reaction parameters and extracting the apparent activation energy (*E*_app_) and apparent pre-exponential factor (*A*_app_) from an Arrhenius plot.

The HER mechanism is assumed to start with a discharge of a proton, *i.e.*, the Volmer step (eqn (7)), followed by an electrochemical step, *i.e.*, Heyrovsky step (eqn (8)) or a chemical recombination step, *i.e.*, the Tafel step (eqn (9)).^42^

7 H^+^ + e^−^ + * ⇌ H_ad_

8 H^+^ + e^−^ + H_ad_ ⇌ H_2_ + *

9 H_ad_ + H_ad_ ⇌ H_2_ + 2*

We distinguish between these two cases in which molecular hydrogen is formed, either through the Heyrovsky step (Volmer–Heyrovsky mechanism, eqn (7) and (8)) or the Tafel step (Volmer–Tafel mechanism, eqn (7) and (9)). The rate equations describing the kinetics of the Volmer–Heyrovsky and Volmer–Tafel are given in eqn (10) and (11), respectively.^43^10
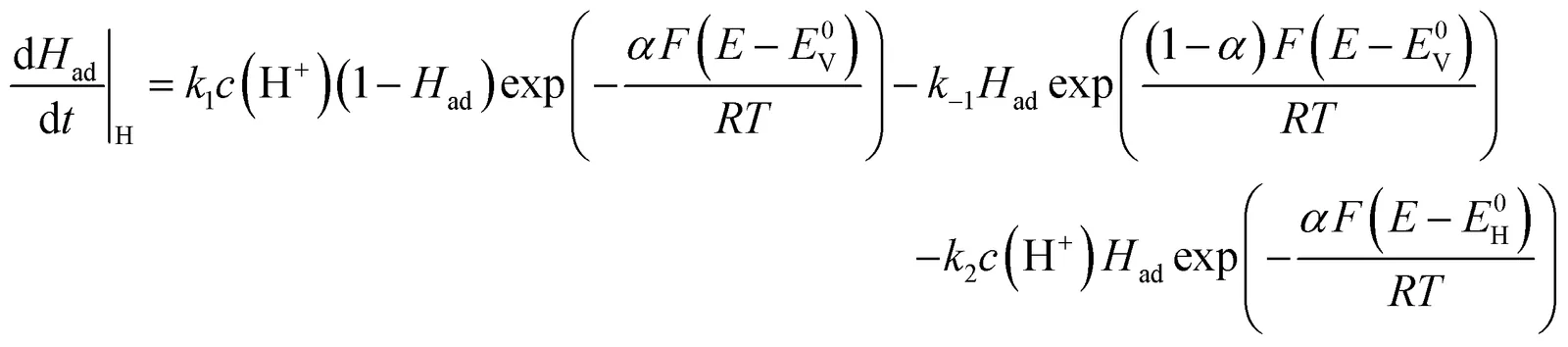
11
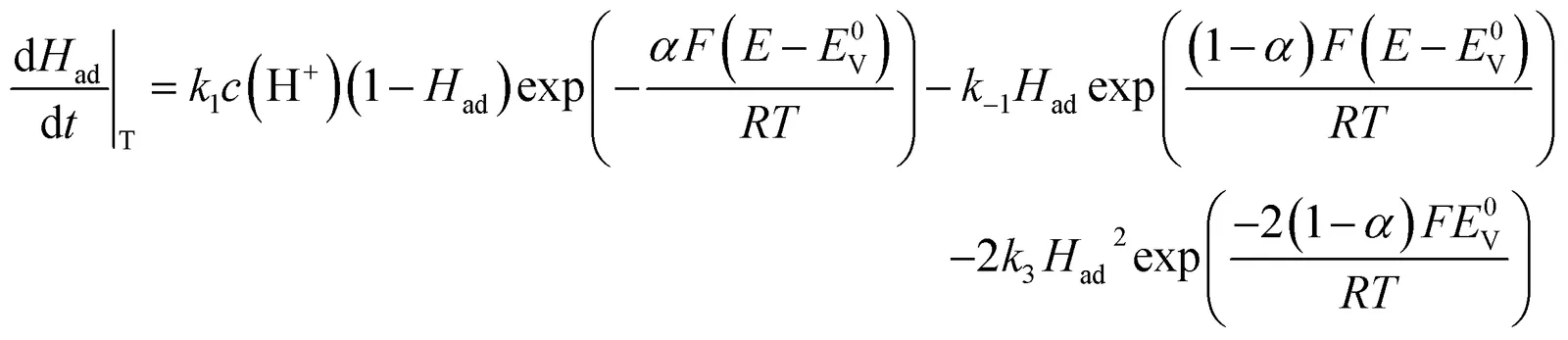


*k*_1_, *k*_−1_, are the forward and backward standard rate constants of the Volmer step. *k*_2_ and *k*_3_ are the forward (standard) rate constants of the Heyrovsky and Tafel steps. Backward rates of the Heyrovsky and Tafel steps are not considered. *H*_ad_ is the coverage of adsorbed hydrogen, *c*(H^+^) is the concentration of protons in solution, *α* is the transfer coefficient, *E*^0^_v_ and *E*^0^_H_ are the equilibrium potentials of the Volmer and Heyrovsky step, respectively, which are defined by the binding energy of adsorbed hydrogen.^44^*E* is the electrode potential *vs.* the standard hydrogen electrode (SHE), *T* is the temperature, *R* is the molar gas constant and *F* is Faraday’s constant. We set the concentration of protons to 1 M, the transfer coefficient to 0.5 and the summation of the Volmer and Heyrovsky equilibrium potentials is fixed to 0 V.

To obtain an analytical solution, we assume the reaction is in a steady state in which the coverage of adsorbed hydrogen does not change with time. Setting eqn (10) and (11) equal to zero, we obtain the expressions for the coverages in each case:12
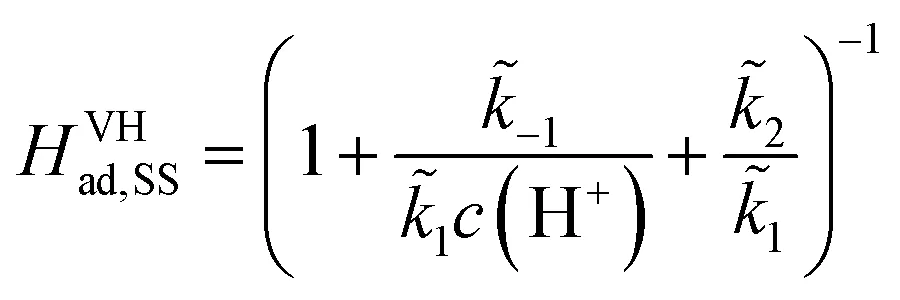
13
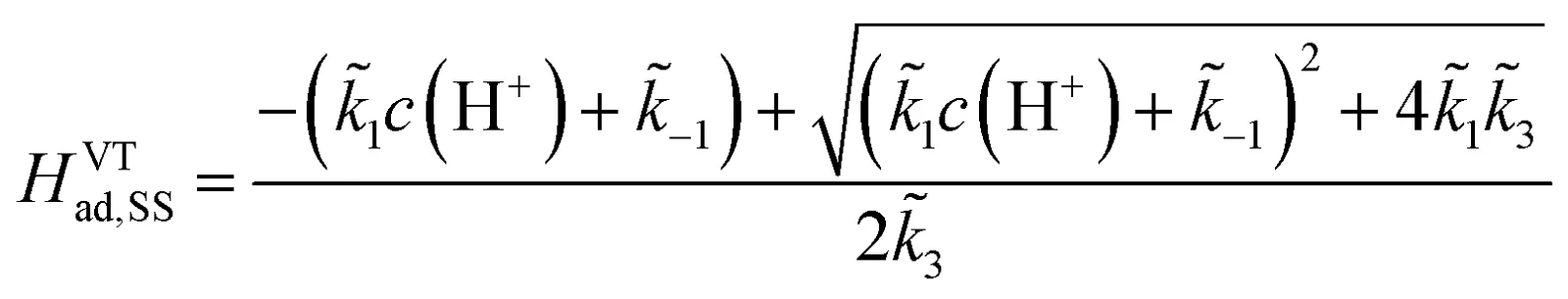


For convenience, we summarized *k*_1_, *k*_−1_, *k*_2_ and *k*_3_ and their respective exponential expressions in eqn (12) and (13) as 
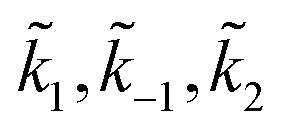
 and 
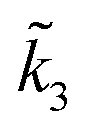
, respectively, *i.e.*
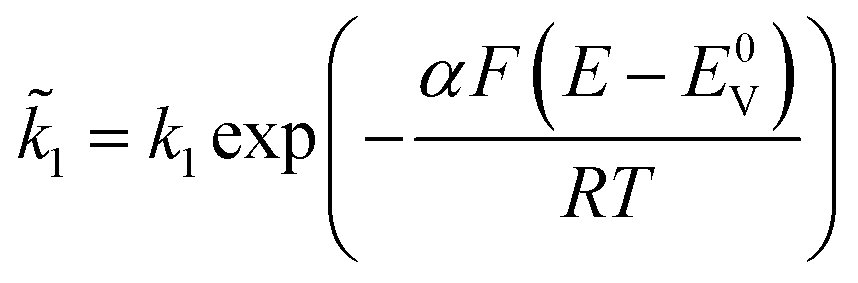
*etc.*

The steady state currents of the Volmer–Heyrovsky and the Volmer–Tafel mechanism are computed according to the following equations14
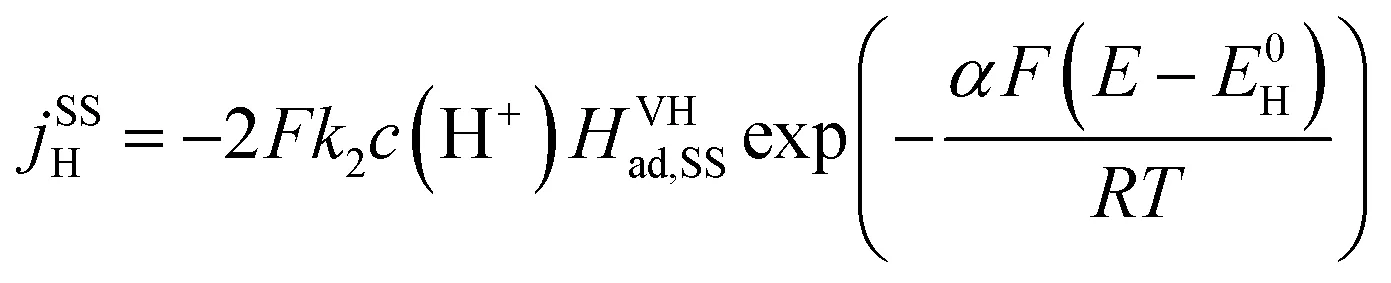
15
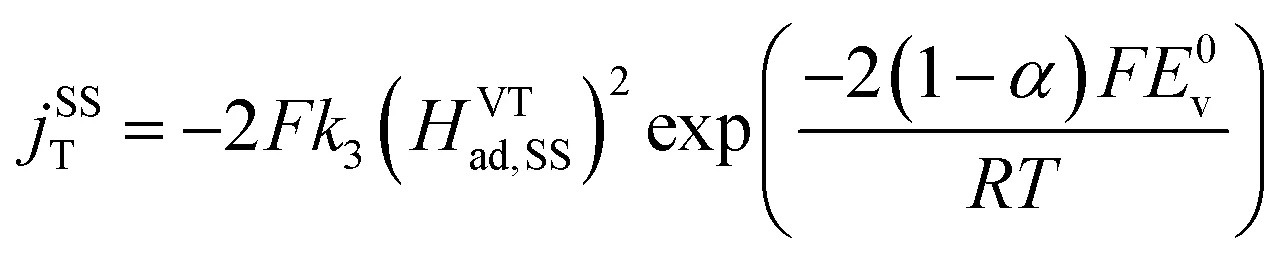


The above expressions are used to generate steady state currents as a function of temperature (298 to 343 K) and overpotential (−0.20 to −0.01 V). We use the equilibrium potentials of the Volmer and Heyrovsky step (*E*^0^_V_ and *E*^0^_H_, respectively) as variable input parameters to change the Gibbs free-energy of the two separate steps at zero overall overpotential. This allows us to study the effect of a change in hydrogen coverage on the observed kinetics. The input parameters for the different model outcomes are summarized in Table S1 in the SI. For convenience, and if not stated otherwise, we set the pre-exponential factor of all elementary steps per default to *k*^0^_*j*_ = 10^13^.^45^ Because our equations are formulated using fractional coverages, the units of the rate constant of the Volmer forward and Heyrovsky forward reaction are l s^−1^ mol^−1^, for the other steps s^−1^. This allows us to use the standard Gibbs free activation energies as the major parameters to generate different scenarios by making different elementary steps rate-determining. As shown in eqn (12) and (13), the hydrogen coverage depends on the ratios of the pre-exponential factors, *k*^0^_*j*_, not on their absolute magnitudes. Apparent Arrhenius parameters (log_10_(*A*_app_), *E*_app_) are extracted by linear regression of ln(|*j*^ss^|) *vs.* 1/*T* and are plotted in a Constable plot, *i.e.* log_10_(*A*_app_) *vs. E*_app_.^46^ Tafel slopes are extracted by linear regression of log_10_|*j*^ss^| *vs.* overpotential.

## Results

### Single active site

**Fig. 1:**
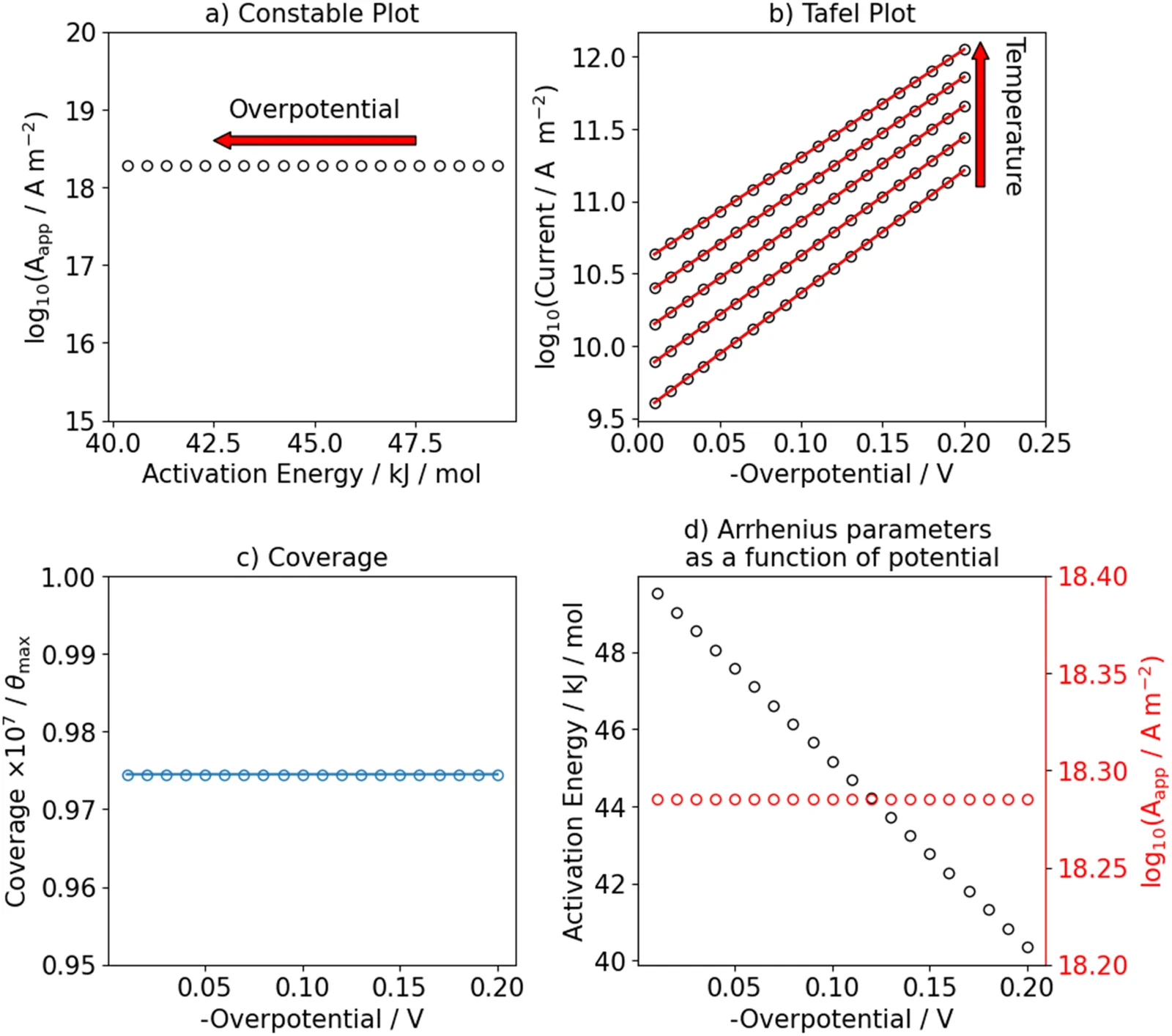
Numerical results of the steady state current of the HER assuming the Volmer–Heyrovsky mechanism. The Volmer step is assumed to be rate-determining by setting Δ*G*^‡^_act,V_ ≫ Δ*G*^‡^_act,H_, namely Δ*G*^‡^_act,V_ = 50 kJ mol^−1^ and Δ*G*^‡^_act,H_ = 10 kJ mol^−1^. The equilibrium potential of the Volmer step is set to *E*^0^_V_ = 0 V. Panel (a) shows the resulting Constable plot. The arrow indicates the change of the apparent Arrhenius parameter as a function of applied potential. Panel (b) shows the corresponding Tafel plot. The Tafel slope at 298 K is 118 mV dec^−1^. Panel (c) shows the steady state coverage of hydrogen in the investigated potential range at 298 K. Panel (d) shows the apparent Arrhenius parameters (activation energy (black) and pre-exponential factor (red)) as a function of applied potential.

We start by showing limiting cases in which a single elementary reaction is assumed to be rate-determining. First, we consider the scenario in which the Volmer step is rate-determining by setting its standard Gibbs free activation energy significantly higher than for the Heyrovsky step (Δ*G*^‡^_0,V_ ≫ Δ*G*^‡^_0,H_, Δ*G*^‡^_0,V_ − Δ*G*^‡^_0,H_ = 40 kJ mol^−1^). The results are shown in Fig. 1. Under these conditions, the steady state coverage of hydrogen is approximately zero over the investigated temperature and potential range, see Fig. 1c. These results are not influenced by the equilibrium potential of the Volmer step (and consequently, of the Heyrovsky step) as long as Δ*G*^‡^_0,V_ ≫ Δ*G*^‡^_0,H_. As expected from simple Butler–Volmer kinetics, the apparent activation energy of the HER decreases as a function of applied (negative) overpotential, as shown in Fig. 1d and as indicated by the red arrow in Fig. 1a. The Tafel slope at room temperature is 118 mV dec^−1^ and constant in the investigated potential range, see Fig. 1b and S5. In this case, an Arrhenius plot over an extended temperature range is linear as shown in Fig. S1.

**Fig. 2:**
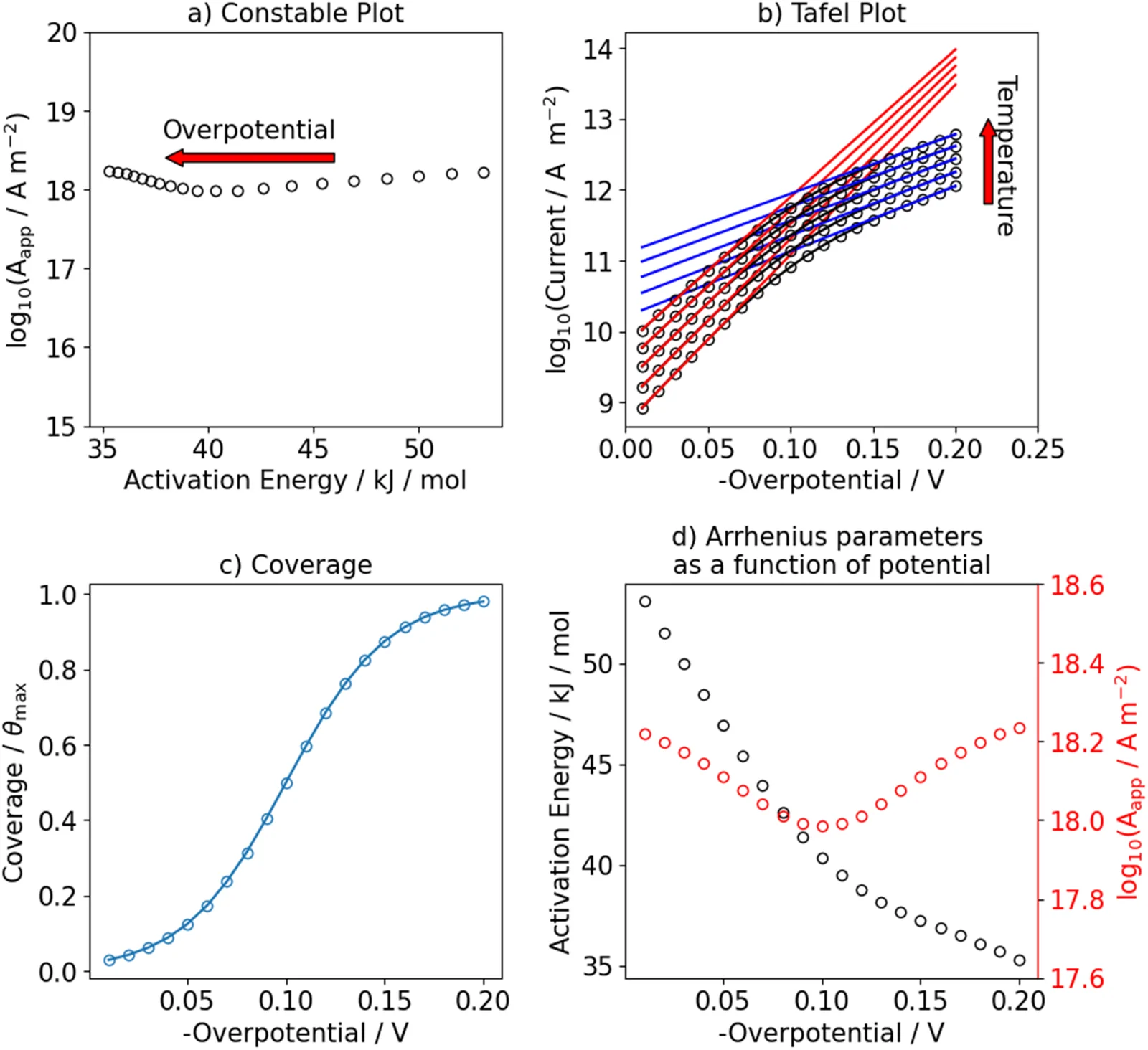
Numerical results of the steady state current of the HER assuming the Volmer–Heyrovsky mechanism. The Heyrovsky step is assumed to be rate-determining by setting Δ*G*^‡^_act,V_ ≪ Δ*G*^‡^_act,H_, namely Δ*G*^‡^_act,V_ = 10 kJ mol^−1^ and Δ*G*^‡^_act,H_ = 50 kJ mol^−1^. The equilibrium potential of the Volmer step is set to *E*^0^_V_ = −0.1 V. Panel (a) shows the resulting Constable plot. The standard activation energy of the Heyrovsky step is set to 50 kJ mol^−1^. The arrow indicates the change of the apparent Arrhenius parameter as a function of applied potential. Panel (b) shows the corresponding Tafel plot. The Tafel slope at 298 K is 40 mV dec^−1^ above 0.1 V and 118 mV dec^−1^ below. The red and blue solid lines show fits to the corresponding regions. Panel (c) shows the steady state coverage of hydrogen in the investigated potential range at 298 K. Panel (d) shows the apparent Arrhenius parameters (activation energy (black) and pre-exponential factor (red)) as a function of applied potential.

Next, we investigated the effect of a change in hydrogen coverage as function of potential on the apparent Arrhenius parameters by making the Heyrovsky step the RDS. The equilibrium potential of the Volmer step is set to −0.1 V and hydrogen coverage increases from zero to full saturation over the studied potential range. This causes a change in the Tafel slope as shown in Fig. 2b from 40 mV dec^−1^ at low overpotentials to 118 mV dec^−1^ at high overpotentials. Additionally, we show in Fig. S7 the potential dependence of the Tafel slope. Interestingly, this also leads to an apparent compensation effect in the Constable plot as shown in Fig. 2a. Although the overall trend follows the behavior expected from BV kinetics, the apparent pre-exponential factor and activation energy both decrease as the potential is decreased from −0.01 V to −0.10 V. At potentials more negative than −0.1 V, the apparent activation energy decreases with a different slope, while the apparent pre-exponential factor increases and eventually reaches the same value as at low overpotentials. We investigated the origin of this apparent compensation effect in more detail in Fig. S3. In short, we observe that the increase in hydrogen coverage with overpotential and the decrease in the activation Gibbs free-energy of the Heyrovsky step leads to a change in the kinetic regime. At low overpotentials, the reaction can be described with a pre-equilibrium situation between protons and adsorbed hydrogen while it changes to a sequential two-step process at higher overpotentials. Note that we have also investigated the scenario where the Heyrovsky step is the RDS but with an equilibrium potential of the Volmer step of 0.1 V, see Fig. S2. In this case, the observed effects are very similar to what we have shown in Fig. 1 because the hydrogen coverage is almost constant in the potential range of interest.

**Fig. 3:**
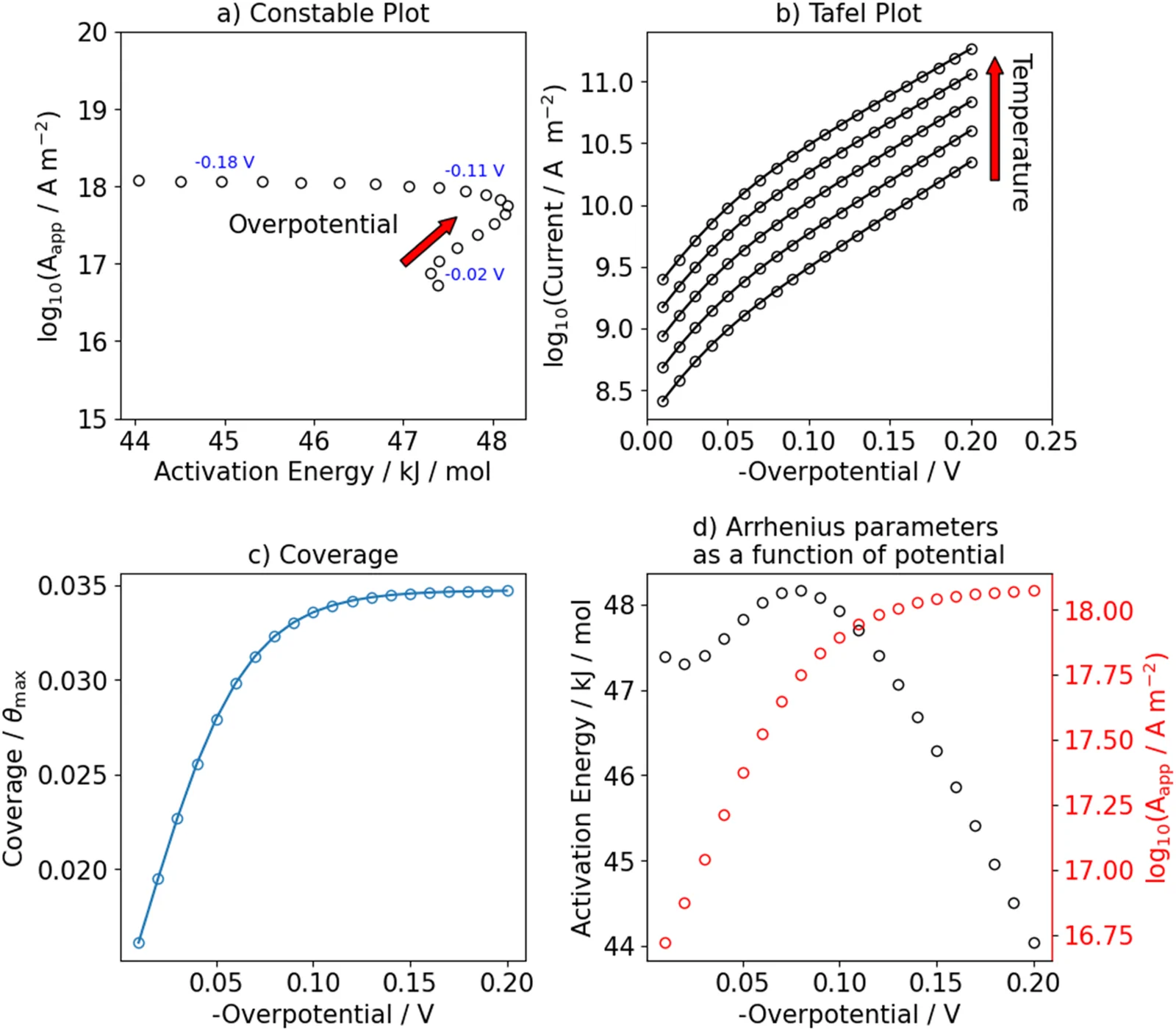
Numerical results of the steady state current of the HER assuming the Volmer–Heyrovsky mechanism. The difference between the activation energy of the Volmer and Heyrovsky step is set to: Δ*G*^‡^_act,V_ − Δ*G*^‡^_act,H_ = 10 kJ mol^−1^ and the ratio of the pre-exponential factors to: 
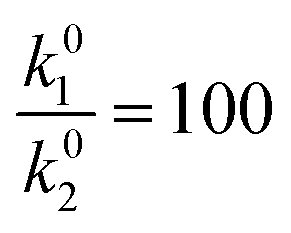
 The equilibrium potential of the Volmer step is set to *E*^0^_V_ = −0.1 V. Panel (a) shows the resulting Constable plot. The arrow indicates the change of the apparent Arrhenius parameter as a function of applied potential. Panel (b) shows the corresponding Tafel plot. At low overpotentials, the Tafel slope is not constant but as the hydrogen coverage saturates, the Tafe slope approaches 118 mV dec^−1^. Panel (c) shows the steady state coverage of hydrogen in the investigated potential range at 298 K. Panel (d) shows the apparent Arrhenius parameters (activation energy (black) and pre-exponential factor (red)) as a function of applied potential.

**Fig. 4:**
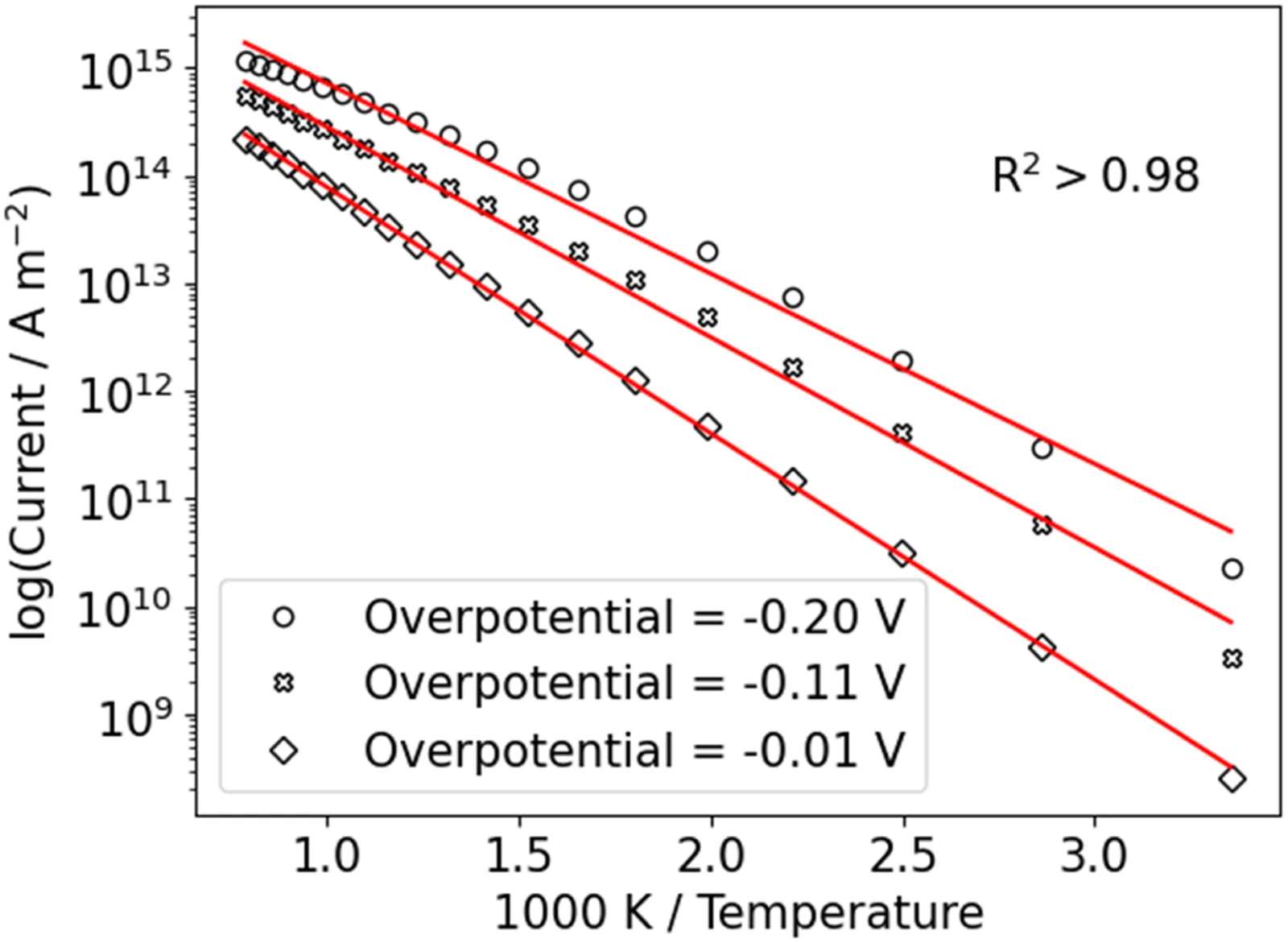
Arrhenius plot of the current of the Volmer–Heyrovsky mechanism over a temperature range from 298 K to 1073 K for three overpotentials (see legend) is shown. The difference between the activation energy of the Volmer and Heyrovsky step is set to: Δ*G*^‡^_act,V_ − Δ*G*^‡^_act,H_ = 10 kJ mol^−1^ and the ratio of the pre-exponential factors to: 
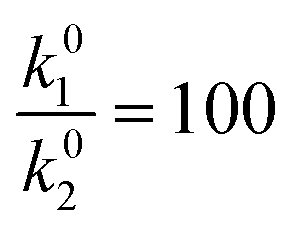
 The equilibrium potential of the Volmer step is set to *E*^0^_V_ = −0.1 V. The *R*^2^-values are larger than 0.98 but importantly, the Arrhenius plot shows a curvature which indicates a deviation of the effective rate law from pure Arrhenius behavior.

We use this rationale to create a case where initially not a single reaction is the RDS and where the coverage of hydrogen changes in the investigated potential window by setting *E*^0^_V_ to −0.1 V. The ratio of the pre-exponential factors, *k*^0^_*i*_ of the Volmer and Heyrovsky steps is set to 
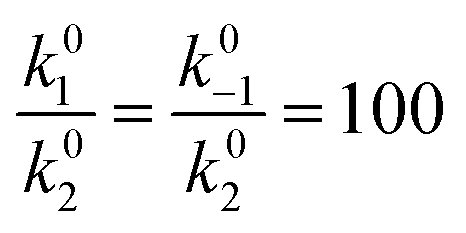
 and the difference of the activation Gibbs free-energy to Δ*G*^‡^_0,V_ − Δ*G*^‡^_0,H_ = 10 kJ mol^−1^. These values are within the expected variation of Arrhenius parameters for the HER.^47–49^ Especially at low overpotentials, the Tafel slope is not constant, but shows a continuous curvature, as shown in Fig. 3b and S8. Additionally, we see a complex compensation effect in the Constable plot. At low overpotentials, both the pre-exponential factor and the activation energy increase while the steady state coverage of hydrogen on the catalyst increases. As soon as the steady state coverage approaches its saturation value, the apparent activation energy decreases as function of overpotential while the pre-exponential factor remains constant. As discussed before, this compensation effect is caused by a similar interplay between the individual rates of the depleting reactions that change with overpotential. Moreover, as shown in Fig. 4, the Arrhenius plot over an extended temperature range shows a clear curvature, indicating a change in the effective rate law, which is indicated by an *R*^2^ value of about 0.98. However, when a smaller temperature range from 298 K to 343 K is considered, one would still obtain an *R*^2^ value of better than 0.9999.

**Fig. 5:**
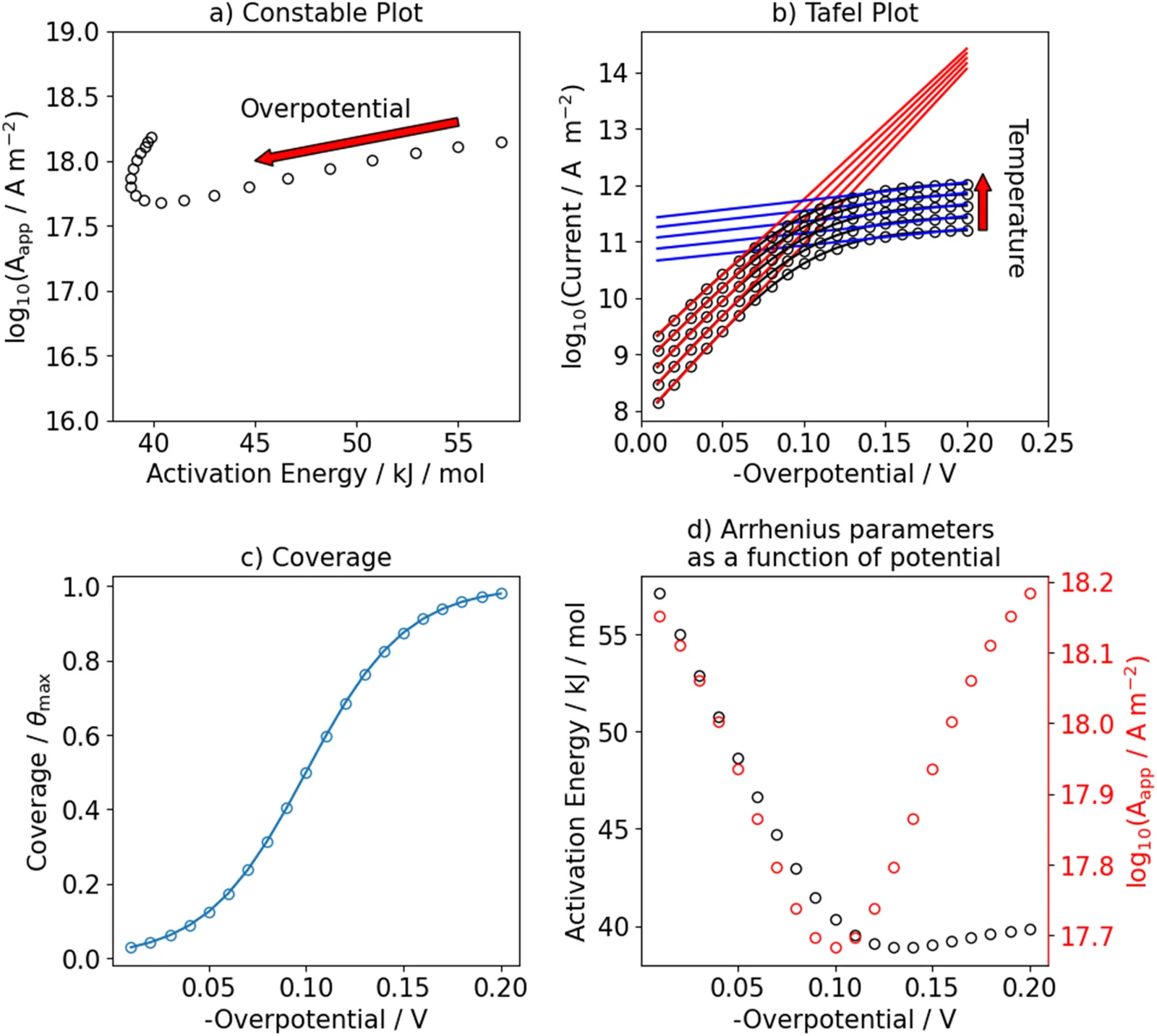
Numerical results of the steady state current of the HER assuming the Volmer–Tafel mechanism. The Tafel step is rate-determining by setting Δ*G*^‡^_act,V_ ≪ Δ*G*^‡^_act,T_, namely Δ*G*^‡^_act,V_ = 10 kJ mol^−1^ and Δ*G*^‡^_act,T_ = 50 kJ mol^−1^. The equilibrium potential of the Volmer step is set to *E*^0^_V_ = −0.1 V. Panel (a) shows the resulting Constable plot. The arrow indicates the change of the apparent Arrhenius parameter as a function of applied potential. Panel (b) shows the corresponding Tafel plot. The Tafel slope at 298 K above −0.06 V is extrapolated to 30 mV dec^−1^ and approaches infinity below. Panel (c) shows the steady state coverage of hydrogen in the investigated potential range at 298 K. Panel (d) shows the apparent Arrhenius parameters (activation energy (black), pre-exponential factor (red)) as a function of applied potential.

Similar cases can be created for the Volmer–Tafel mechanism. We make the same observation in the Constable and Tafel plot for the Volmer–Tafel mechanism when the Volmer step is the RDS, see Fig. S4 and S9. We observe new features in the Constable plot when the Tafel step is the RDS of the reaction. In Fig. 5, the equilibrium potential of the Volmer-step is set to −0.1 V to investigate the effect of a change in steady state coverage on the apparent activation energy and pre-exponential factor. Both parameters first decrease at more positive potentials than −0.1 V and then increase as we move to higher (more negative) overpotentials. This occurs concurrently with a change in the Tafel slope as shown in Fig. 5b and S10. Note the difference in the potential dependence observed in the Constable plot when the Heyrovsky step is rate-determining (Fig. 2a), showing a small dip with increasing potential, compared to when the Tafel step is rate-determining (Fig. 5a) showing a clear compensation effect which changes at larger overpotentials. We currently do not have enough numerical examples to judge whether these are general features, but clearly a more detailed numerical and analytical study on the relation between the shape and features of the Constable plot and the (electro)catalytic mechanism will be of great interest.

### Multiple active sites

We also consider the scenario in which two active sites exist on the electrocatalyst. We assume the same Arrhenius parameters between the two sites, but vary the equilibrium potential of the Volmer step and consequently that of the Heyrovsky step. We take the sum of steady state currents of the individual sites, *j*^SS^_total_, and apply the same analysis as before.

**Fig. 6:**
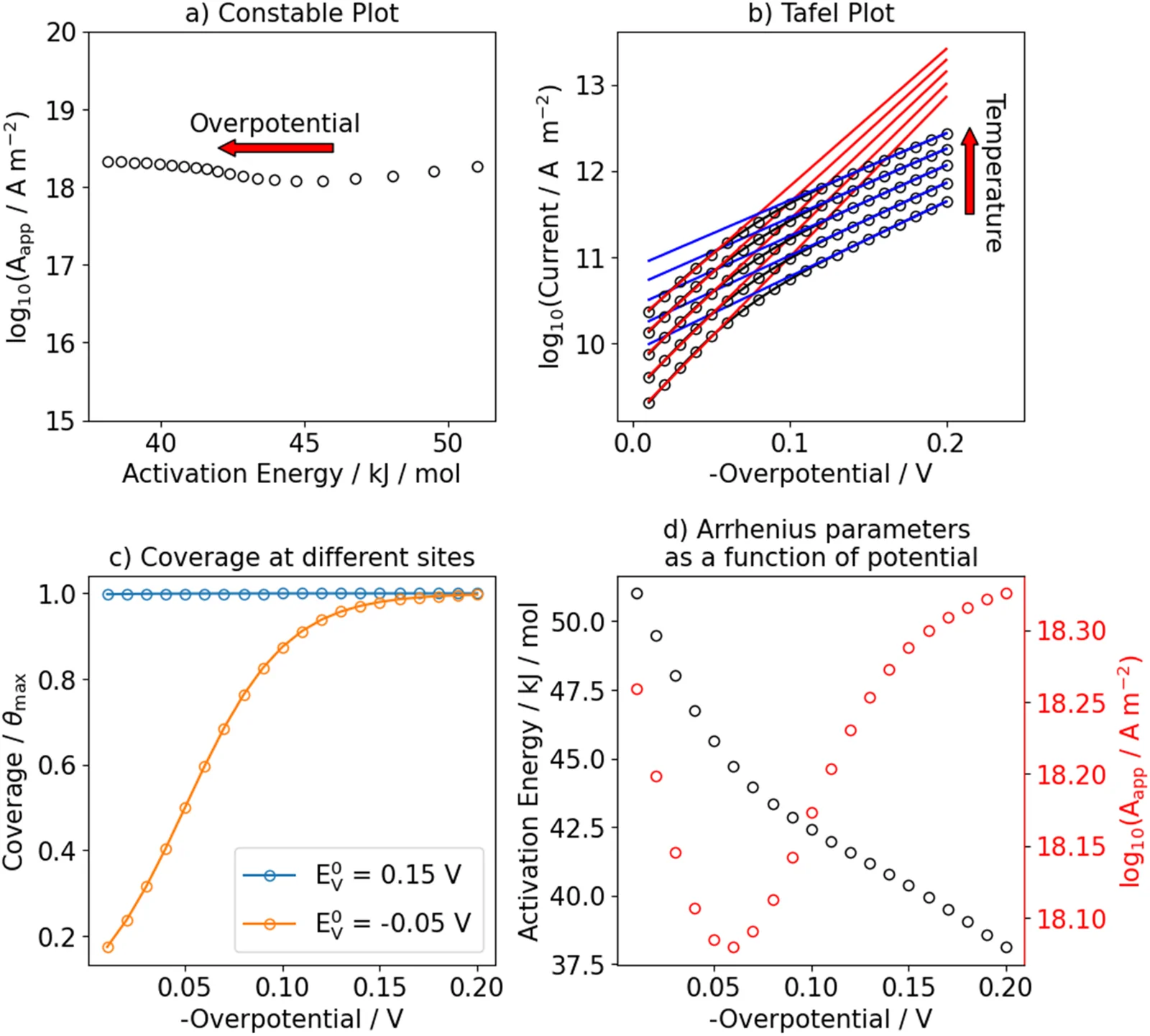
Numerical results of multiple sites contributing to the total kinetic current of the HER assuming the Volmer–Heyrovsky mechanism. The Heyrovsky step is rate-determining by setting Δ*G*^‡^_act,V_ ≪ Δ*G*^‡^_act,H_, namely Δ*G*^‡^_act,V_ = 10 kJ mol^−1^ and Δ*G*^‡^_act,H_ = 50 kJ mol^−1^. The equilibrium potentials of the Volmer step at the two different sites are set to *E*^0^_V,1_ = 0.15 V and *E*^0^_V,2_ = −0.05 V. Panel (a) shows the resulting Constable plot. The arrow indicates the change of the apparent Arrhenius parameter as a function of applied potential. Panel (b) shows the corresponding Tafel plot. The Tafel slope at 298 K below −0.1 V approaches 118 mV dec^−1^. Panel (c) shows the coverage of adsorbed hydrogen on the two different sites as a function of potential. Panel (d) shows the apparent Arrhenius parameters (activation energy (black), pre-exponential factor (red)) as a function of applied potential. Fig. S11 shows the Tafel slope as a function of overpotential.

Fig. 6 shows an example for a catalyst which consists of two sites on which the HER occurs through the Volmer–Heyrovsky mechanism, but with different equilibrium potentials for the Volmer step (*E*^0^_V,1_ = 0.15 V and *E*^0^_V,2_ = −0.05 V). On both sites the Heyrovsky-step is rate-determining. This leads to a very similar situation to that shown in Fig. 2. While one site is already saturated with hydrogen and thus, the apparent activation energy decreases while the pre-exponential factor is constant, the other site starts to get filled with hydrogen in the investigated potential range, which leads to the same trends as discussed before.

**Fig. 7:**
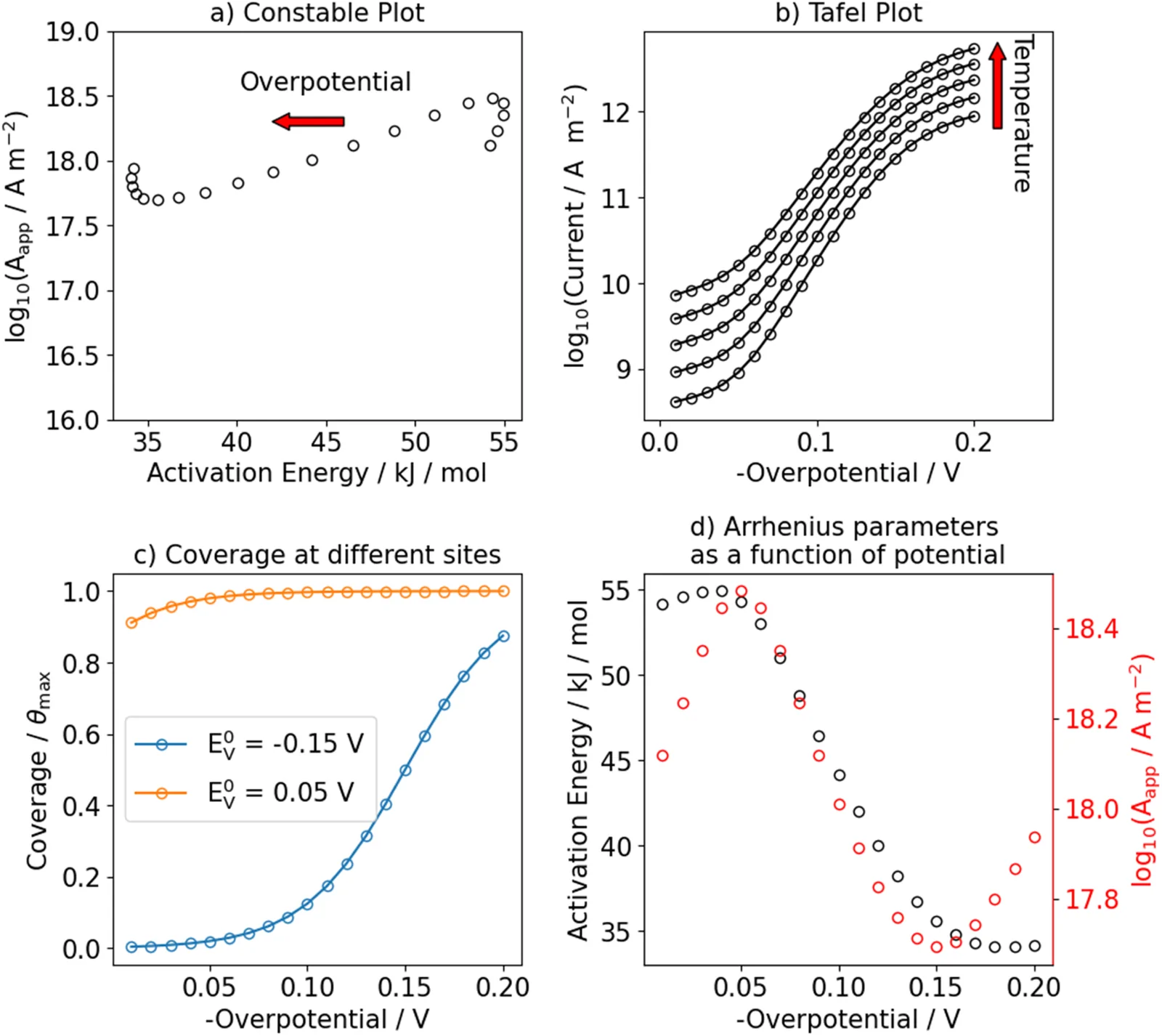
Numerical results of multiple sites contributing to the total kinetic current of the HER assuming the Volmer–Tafel mechanism. The Tafel step is rate-determining by setting Δ*G*^‡^_act,V_ ≪ Δ*G*^‡^_act,T_, namely Δ*G*^‡^_act,V_ = 10 kJ mol^−1^ and Δ*G*^‡^_act,T_ = 50 kJ mol^−1^. The equilibrium potentials of the Volmer step at the two different sites are set to *E*^0^_V,1_ = −0.15 V and *E*^0^_V,2_ = 0.05 V. Panel (a) shows the resulting Constable plot. The arrow indicates the change of the apparent Arrhenius parameter as a function of applied potential. Panel (b) shows the corresponding Tafel plot. The Tafel slope is not constant over the investigated potential window. Panel (c) shows the coverage of adsorbed hydrogen on the two different sites as a function of potential. Panel (d) shows the apparent Arrhenius parameters (activation energy (black), pre-exponential factor (red)) as a function of applied potential. Fig. S12 shows the Tafel slope as a function of the overpotential.

If we assume two sites, but molecular hydrogen is formed through the Volmer–Tafel mechanism, we obtain results as shown in Fig. 7. In subpanel (a), at low overpotentials the previously discussed signature of the Tafel-step – where the apparent activation energy and pre-exponential factor increase with applied overpotential – is observed. Around the equilibrium potential of the Volmer-step of the second site, this site starts to get covered in hydrogen, which results in a decrease of both Arrhenius parameters. At high enough overpotentials, the second site also approaches saturation coverage and the same increase of apparent activation energy and pre-exponential factor is observed.

## Discussion and conclusions

The simulation results in the previous sections demonstrate that even in ideally chosen systems (free of heterogeneity, no intrinsic potential-dependent activation entropy in the elementary reaction rates, no competing reactions from impurities, *etc.*) an apparent compensation effect can arise from changes in the kinetic regime as a function of electrode potential. Mathematically, these effects arise from the effective rate law not exactly satisfying (the temperature dependence of) the Arrhenius law. Moreover, in the case of a “heterogeneous” surface, modelled here as two sites differing in activity, a curved Tafel plot, *i.e.* with no single Tafel slope, can be obtained, and an apparent compensation effect is observed. In cases where such phenomena are observed *i.e.*, changes between constant Tafel slopes and/or potential-dependent Tafel slopes (*i.e.*, no single Tafel slope value), we conclude that it cannot unequivocally be demonstrated that the observed deviations from ideality are due to a single factor, *i.e.*, potential-dependent changes in activation entropy in elementary reaction steps.

While most studies on the temperature dependence of the HER have focused on polycrystalline materials, where deconvoluting facet-dependent adsorbate and surface charge contributions are intractable, a compensation effect has been shown to arise for more “idealized” electrode materials such as Hg and single-crystalline metal electrodes (*i.e.*, Ag and Pt). Schmickler and colleagues studied the temperature dependence of the HER on both Ag and Pt single-crystal electrodes and observed non-idealities (*i.e.*, increasing apparent activation energy with more negative potentials and a potential dependent pre-exponential factor, quite similar to what we modeled in Fig. 3), which for the case of Ag they tentatively attributed to the effect to adsorption of anions.^10^ For the case of a Pt(111) single-crystal electrode, they observed a curved Tafel plot, which as discussed above, makes the attribution of the observed compensation effect challenging.^9^ Defects inherently found on Pt(111) may show high HER activity compared to the terrace atoms and may be the cause of the deviation from ideality.^50,51^ Also note that from plots such as shown in Fig. 2d, 3d, 5d and 7d, one could derive enthalpic and entropic contributions to the transfer coefficient, as originally defined by Conway.^18,21^ However, those quantities would not have a clear physical meaning, as they would, again, be a result from fitting a non-Arrhenius rate law by the Arrhenius law. Hg would by far be the most ideal material for studying fundamental concepts such as the compensation effect, as it reduces the side phenomena that may cause an apparent compensation effect. Conway indeed observed a compensation effect when studying the HER on Hg in acidic media in a potential regions where a constant Tafel slope is observed (*i.e.*, ensuring that there is no change in kinetic regimes).^17,52^ Nonetheless, the apparent compensation effect for this system was later shown to arise from the diffuseness of the double layer and when accounting for it, no compensation effect was observed.^35,53^

In general, linking an observed compensation effect in electrochemical measurements to intrinsic entropic contributions is difficult due to the high complexity of the electrode–electrolyte interface, which will undermine the assumption that the reaction rate will exactly satisfy the Arrhenius law at every potential. Here, we have shown even for a prototypical, simple electrochemical reaction that a compensation effect can occur when: (1) there is a change in the rate-determining step or a change in balance between two steps, as a function of potential; (2) multiple active sites are present on a catalyst. We have shown that in both cases, non-constant Tafel slopes indicate the concurrence of a compensation effect. In general, interpretation of observed compensation effects should be done with caution, especially when a multi-step reaction is investigated as a function of potential and temperature. Both parameters can change the effective rate law – resulting in a curved Arrhenius plot – and cause an apparent compensation effect which does not signify any intrinsic entropy–enthalpy compensation. As outlined by others before, in such cases the observed compensation effect has a mathematical, *i.e.* non-intrinsic, origin.^26,27,54^

Regardless of our results and conclusions based on these simple models, studying the compensation effect for electrochemical reactions is a fascinating topic that might be able to resolve some of the currently unanswered questions about mechanisms and interfacial electrolyte effects. Apart from a more detailed numerical and analytical study of different models, we propose that these insights should preferably be tested against experiments using well-defined single crystal electrodes in potential regions where the Tafel slope is sufficiently constant.

## Author contributions

Conceptualization: R. E. V., O. v. H. and M. T. M. K. Data curation: J. F. Formal analysis: J. F., R. Z. S. and M. A. Funding acquisition: J. F., R. Z. S. and M. T. M. K. Investigation: J. F., R. Z. S., M. A., R. E. V., O. v. H. and M. T. M. K. Methodology: J. F. and R. Z. S. Project administration: M. T. M. K. Software: J. F. and R. Z. S. Supervision: M. T. M. K. Validation: J. F., R. Z. S., M. A., R. E. V., O. v. H. and M. T. M. K. Visualization: J. F. and R. Z. S. Writing – original draft: J. F., R. Z. S., M. A., and M. T. M. K. Writing – review & editing: J. F., R. Z. S., M. A., R. E. V., O. v. H. and M. T. M. K.

## Conflicts of interest

The authors declare no conflict of interest.

## Supplementary Material

FD-267-D5FD00163C-s001

## Data Availability

Our model, the input parameters and figures are summarized in a jupyter-notebook that can be accessed through https://github.com/JanFingerhut/VHTmechanism. The data supporting this article have been included as part of the supplementary information (SI). Supplementary information is available. See DOI: https://doi.org/10.1039/d5fd00163c.

## References

[cit1] Bard A. J. , Faulkner L. R. and White H. S., Electrochemical Methods: Fundamentals and Applications, John Wiley & Sons, 2022

[cit2] Lee K.-G., Balamurugan M., Park S., Ha H., Jin K., Seo H., Nam K. T. (2019). Importance of Entropic Contribution to Electrochemical Water Oxidation Catalysis. ACS Energy Lett..

[cit3] Martínez-Hincapié R., Timoshenko J., Wagner T., Ortega E., Druce J., Monteiro M. C. O., Rüscher M., Jang J., Alagöz E. Ö., Lasagna S., Jacobse L., Bergmann A., Cuenya B. R., Oener S. Z. (2025). Interfacial solvation pre-organizes the transition state of the oxygen evolution reaction. Nat. Chem..

[cit4] Gisbert-González J. M., Rodellar C. G., Druce J., Ortega E., Cuenya B. R., Oener S. Z. (2025). Bias Dependence of the Transition State of the Hydrogen Evolution Reaction. J. Am. Chem. Soc..

[cit5] Rodellar C. G., Gisbert-Gonzalez J. M., Sarabia F., Roldan Cuenya B., Oener S. Z. (2024). Ion solvation kinetics in bipolar membranes and at electrolyte–metal interfaces. Nat. Energy.

[cit6] Zeradjanin A. R., Narangoda P., Masa J., Schlögl R. (2022). What Controls Activity Trends of Electrocatalytic Hydrogen Evolution Reaction?—Activation Energy Versus Frequency Factor. ACS Catal..

[cit7] Zeradjanin A. R., Narangoda P., Spanos I., Masa J., Schlögl R. (2021). Expanding the frontiers of hydrogen evolution electrocatalysis–searching for the origins of electrocatalytic activity in the anomalies of the conventional model. Electrochim. Acta.

[cit8] Conway B., Tessier D., Wilkinson D. (1989). Temperature dependence of the Tafel slope and electrochemical barrier symmetry factor, β, in electrode kinetics. J. Electrochem. Soc..

[cit9] He Z.-D., Wei J., Chen Y.-X., Santos E., Schmickler W. (2017). Hydrogen evolution at Pt(111) – activation energy, frequency factor and hydrogen repulsion. Electrochim. Acta.

[cit10] He Z.-D., Chen Y.-X., Santos E., Schmickler W. (2018). The Pre-exponential Factor in Electrochemistry. Angew. Chem., Int. Ed..

[cit11] Eberhardt D., Santos E., Schmickler W. (1999). Hydrogen evolution on silver single crystal electrodes—first results1Dedicated to Professor W. Vielstich on the occasion of his 75th birthday.1. J. Electroanal. Chem..

[cit12] Tamm J., Tamm L., Vares P. (2000). Temperature dependence of hydrogen overvoltage on nickel and iron in acid solution. Russ. J. Electrochem..

[cit13] van der Heijden O., Vos R. E., Koper M. T. M. (2025). Temperature-Dependent Kinetic Parameters for the Alkaline Oxygen Evolution Reaction on NiFeOOH. ACS Energy Lett..

[cit14] Bligaard T., Honkala K., Logadottir A., Nørskov J. K., Dahl S., Jacobsen C. J. H. (2003). On the Compensation Effect in Heterogeneous Catalysis. J. Phys. Chem. B.

[cit15] Boudart M. and Djéga-Mariadassou G., Kinetics of Heterogeneous Catalytic Reactions, Princeton University Press, 1984

[cit16] Chorkendorff I. and Niemantsverdriet J. W., Concepts of Modern Catalysis and Kinetics, John Wiley & Sons, 2017

[cit17] Conway B. E., Wilkinson D. F. (1986). Entropic and enthalpic components of the symmetry factor for electrochemical proton transfeJ. Phys. Chem. Br from various proton donors over a wide temperature range. J. Electroanal. Chem. Interfacial Electrochem..

[cit18] Conway B., MacKinnon D. (1969). Interpretation and significance of heats of activation for electrochemical reactions exhibiting anomalous tafel slopes. J. Electrochem. Soc..

[cit19] Conway B. E., MacKinnon D. J., Tilak B. V. (1970). Significance of electrochemical Brønsted factors. Kinetic studies over a wide range of temperatures. Trans. Faraday Soc..

[cit20] Ulstrup J. (1984). Temperature dependence of the transfer coefficient in electron and atom group transfer processes. Electrochim. Acta.

[cit21] Conway B. E. , The temperature and potential dependence of electrochemical reaction rates, and the real form of the Tafel equation, in Modern Aspects of Electrochemistry No. 16, ed. B. E. Conway, R. E. White and J. O’.M. Bockris, Plenum Publishing Corp, New York and London, 1985

[cit22] Koper M. T. M. (1997). Temperature Dependence of the Transfer Coefficient of Simple Electrochemical Redox Reactions Due to Slow Solvent Dynamics. J. Phys. Chem. B.

[cit23] Bockris J. O. M., Gochev A. (1986). Temperature dependence of the symmetry factor in electrode kinetics. J. Phys. Chem..

[cit24] Schmickler W. (1990). The transfer coefficient in proton transfer reactions. J. Electroanal. Chem. Interfacial Electrochem..

[cit25] Trasatti S. , Reaction mechanism and rate determining steps, in Handbook of Fuel Cells, ed. W. Vielstich, A. Lamm, H. A. Gasteiger and H. Yokokawa, 2010

[cit26] Barrie P. J. (2012). The mathematical origins of the kinetic compensation effect: 1. the effect of random experimental errors. Phys. Chem. Chem. Phys..

[cit27] Barrie P. J. (2012). The mathematical origins of the kinetic compensation effect: 2. the effect of systematic errors. Phys. Chem. Chem. Phys..

[cit28] Bond G. C., Keane M. A., Kral H., Lercher J. A. (2000). Compensation Phenomena in Heterogeneous Catalysis: General Principles and a Possible Explanation. Catal. Rev..

[cit29] Kriksunov L. B. (1995). The temperature dependence of the Tafel slope for parallel electrochemical reactions. Electrochim. Acta.

[cit30] Huang Z., Cheng T., Shah A. H., Zhong G., Wan C., Wang P., Ding M., Huang J., Wan Z., Wang S., Cai J., Peng B., Liu H., Huang Y., Goddard W. A., Duan X. (2024). Edge sites dominate the hydrogen evolution reaction on platinum nanocatalysts. Nat. Catal..

[cit31] Koper M. T. M. (2011). Structure sensitivity and nanoscale effects in electrocatalysis. Nanoscale.

[cit32] Vogt C., Weckhuysen B. M. (2022). The concept of active site in heterogeneous catalysis. Nat. Rev. Chem..

[cit33] Kirowa-Eisner E., Schwarz M., Gileadi E. (1989). The temperature dependence of the Tafel slope—I. Instrumentation, calibration and a study of the reduction of hydroxylamine on the dme. Electrochim. Acta.

[cit34] Schwarz M., Kirowa-Eisner E., Gileadi E. (1993). Temperature dependence of the Tafel slope. The reduction of bromate in alkaline media. J. Electroanal. Chem..

[cit35] Kirowa-Eisner E., Schwarz M., Rosenblum M., Gileadi E. (1995). Temperature dependence of the transfer coefficient for the hydrogen evolution reaction on the DME. J. Electroanal. Chem..

[cit36] Oener S. Z. (2025). Transition state tuning with concentrated electrolytes. Nat. Chem..

[cit37] Zhang H., Raciti D., Hall A. S. (2025). Disordered interfacial H O promotes electrochemical C-C coupling. Nat. Chem..

[cit38] Kosakian A., Secanell M. (2021). Estimating charge-transport properties of fuel-cell and electrolyzer catalyst layers via electrochemical impedance spectroscopy. Electrochim. Acta.

[cit39] Kulikovsky A. (2021). Analysis of proton and electron transport impedance of a PEM fuel cell in H 2/N 2 regime. Electrochem. Sci. Adv..

[cit40] van der Heijden O., Park S., Vos R. E., Eggebeen J. J. J., Koper M. T. M. (2024). Tafel Slope Plot as a Tool to Analyze Electrocatalytic Reactions. ACS Energy Lett..

[cit41] van der Heijden O., Eggebeen J. J. J., Trzesniowski H., Deka N., Golnak R., Xiao J., van Rijn M., Mom R. V., Koper M. T. M. (2024). Li+ Cations Activate NiFeOOH for Oxygen Evolution in Sodium and Potassium Hydroxide. Angew. Chem., Int. Ed..

[cit42] Parsons R. (1958). The Rate of Electrolytic Hydrogen Evolution and the Heat of Adsorption of Hydrogen. Trans. Faraday Soc..

[cit43] Koper M.T. M. (2013). Analysis of electrocatalytic reaction schemes: distinction between rate-determining and potential-determining steps. J. Solid State Electrochem..

[cit44] Koper M. T. M. (2011). Thermodynamic theory of multi-electron transfer reactions: Implications for electrocatalysis. J. Electroanal. Chem..

[cit45] He Z. D., Chen Y. X., Santos E., Schmickler W. (2018). The Pre-exponential Factor in Electrochemistry. Angew. Chem., Int. Ed..

[cit46] Constable F. H. (1925). The mechanism of catalytic decomposition. Proc. R. Soc. London, Ser. A.

[cit47] Conway B. E., Barber J., Morin S. (1998). Comparative evaluation of surface structure specificity of kinetics of UPD and OPD of H at single-crystal Pt electrodes. Electrochim. Acta.

[cit48] He Z. D., Wei J., Chen Y. X., Santos E., Schmickler W. (2017). Hydrogen evolution at Pt(111) - activation energy, frequency factor and hydrogen repulsion. Electrochim. Acta.

[cit49] Markovic N. M., Grgur B. N., Ross P. N. (1997). Temperature-dependent hydrogen electrochemistry on platinum low-index single-crystal surfaces in acid solutions. J. Phys. Chem. B.

[cit50] Pohl M. D., Watzele S., Calle-Vallejo F., Bandarenka A. S. (2017). Nature of Highly Active Electrocatalytic Sites for the Hydrogen Evolution Reaction at Pt Electrodes in Acidic Media. ACS Omega.

[cit51] Strmcnik D., Li D., Lopes P. P., Tripkovic D., Kodama K., Stamenkovic V. R., Markovic N. M. (2015). When Small is Big: The Role of Impurities in Electrocatalysis. Top. Catal..

[cit52] Conway B. E., Tessier D. F., Wilkinson D. P. (1986). Experimental evidence for the potential-dependence of entropy of activation in electrochemical reactions in relations to the temperature-dependence of tafel slopes. J. Electroanal. Chem. Interfacial Electrochem..

[cit53] Tsionskii V. M., Krishtalik L. I., Kriksunov L. B. (1988). Temperature dependence of the transfer coefficient: analysis of experimental data. Electrochim. Acta.

[cit54] Widenhorn R., Rest A., Bodegom E. (2002). The Meyer-Neldel rule for a property determined by two transport mechanisms. J. Appl. Phys..

