# Supplementary material for: Compensation effects between the apparent activation energy and pre-exponential factor in simple models of electrocatalytic hydrogen evolution
Source: Faraday Discuss. 2026 Jan 15. Online ahead of print. doi: 10.1039/d5fd00163c (PMC13127248; doi:10.1039/d5fd00163c)
Supplement: FD-OLF-D5FD00163C-s001 [file FD-OLF-D5FD00163C-s001.pdf]

Supplementary Information (SI) for Faraday Discussions.
This journal is © The Royal Society of Chemistry 2026

Supporting information to

Compensation effects between the apparent activation energy and pre-exponential factor in simple models of electrocatalytic hydrogen evolution

Jan Fingerhut[1,†], Rifael Z. Snitkoff-Sol[1,†] , Maximilian Albers[1], Rafaël E. Vos[2], Onno van der Heijden[2], Marc T. M. Koper[1,*]

[1]Leiden Institute of Chemistry, Leiden University, Einsteinweg 55, 2333 CC Leiden, the Netherlands.

[2]Department of Chemical Engineering and Chemistry, Technical University of Eindhoven, 5600 MB, Eindhoven, the Netherlands

*email: m.koper@chem.leidenuniv.nl

† These Authors contributed equally

Input parameters for different model outcomes.

Table S1: A summary of the input parameters to generate the different model outcomes discussed in this work. k_1^0, k_{-1}^0, k_2^0 *and* k_3^0 *are the pre-exponential factors of the Volmer forward, backwards, Heyrovsky forward and Tafel forward step, respectively.* $\Delta G_{0,V}^{\ddagger}$, $\Delta G_{0,H}^{\ddagger}$ *and* $\Delta G_{0,T}^{\ddagger}$ *are the Gibbs activation free-energy of the Volmer, Heyrovsky and Tafel step, respectively.* E_V^0 *and* E_H^0 *are the equilibrium potentials of the Volmer and Heyrovsky reaction, respectively.*

Volmer-Heyrovsky: Volmer is the RDS, see Figure S1 and S5						
$k_1^0/\frac{l}{s\,mol}$	$k_{-1}^0/\frac{1}{s}$	$\Delta G_{0,V}^{\ddagger}/\frac{kJ}{mol}$	$k_2^0/\frac{l}{s\,mol}$	$\Delta G_{0,H}^{\ddagger}/\frac{kJ}{mol}$	E_V^0/V	E_H^0/V
10^{13}	10^{13}	50	10^{13}	10	0	0
Volmer-Heyrovsky: Heyrovsky is the RDS, see Figure 1 and S6						
10^{13}	10^{13}	10	10^{13}	50	0.1	-0.1
Volmer-Heyrovsky: Heyrovsky is the RDS, see Figure 2 and S7						
10^{13}	10^{13}	10	10^{13}	50	-0.1	0.1
Volmer-Heyrovsky: see Figure 3 and S8						
10^{13}	10^{13}	50	10^{11}	40	-0.1	0.1
Volmer-Tafel: Volmer is the RDS, see Figure S4 and S9						
$k_1^0/\frac{l}{s\,mol}$	$k_{-1}^0/\frac{1}{s}$	$\Delta G_{0,V}^{\ddagger}/\frac{kJ}{mol}$	$k_3^0/\frac{1}{s}$	$\Delta G_{0,T}^{\ddagger}/\frac{kJ}{mol}$	E_V^0/V	–
10^{13}	10^{13}	50	10^{13}	10	0	
Volmer-Tafel: Tafel is the RDS, see Figure 5 and S10						
10^{13}	10^{13}	10	10^{13}	50	-0.1	
Volmer-Heyrovsky: multiple active sites, see Figure 6 and S11						
$k_1^0/\frac{l}{s\,mol}$	$k_{-1}^0/\frac{1}{s}$	$\Delta G_{0,V}^{\ddagger}/\frac{kJ}{mol}$	$k_2^0/\frac{l}{s\,mol}$	$\Delta G_{0,H}^{\ddagger}/\frac{kJ}{mol}$	E_V^0/V	E_H^0/V
10^{13}	10^{13}	50	10^{13}	10	$[0.15, -0.05]$	$[-0.15, 0.05]$
Volmer-Tafel: multiple active sites, see Figure 7 and S12						
$k_1^0/\frac{l}{s\,mol}$	$k_{-1}^0/\frac{1}{s}$	$\Delta G_{0,V}^{\ddagger}/\frac{kJ}{mol}$	$k_3^0/\frac{1}{s}$	$\Delta G_{0,T}^{\ddagger}/\frac{kJ}{mol}$	E_V^0/V	
10^{13}	10^{13}	10	10^{13}	50	$[0.15, 0.05]$	

Extended Arrhenius analysis. The steady-state currents for the Volmer-Heyrovsky mechanism (Volmer-step is the RDS) over an (experimentally not accessible) larger temperature range for three overpotentials are shown, see 2. Even over this increased temperature range, the Arrhenius plot is linear with an R^2-value better than 0.999999.

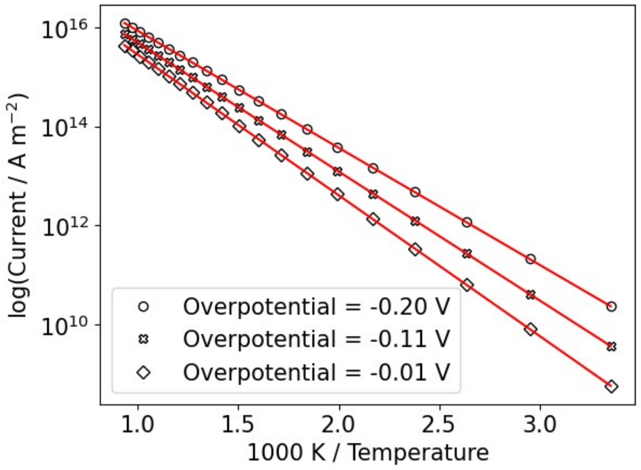

Figure S1: An Arrhenius plot of the current of the Volmer-Heyrovsky mechanism over a temperature range from 298 K to 1073 K for three overpotentials (see legend) is shown. Here, the Volmer step is assumed to be rate-determining by setting the activation Gibbs energy much larger than for the Heyrovsky step, $\Delta G_{act,V}^{\ddagger} \gg \Delta G_{act,H}^{\ddagger}$*. The* R^2*-value is 0.999999.*

Volmer-Heyrovsky mechanism. In Figure S2, we set the Heyrovsky step as the RDS by setting the Gibbs activation free-energy significantly higher than for the Volmer step $\left(\Delta G_{0,V}^{\ddagger} \ll \Delta G_{0,H}^{\ddagger}, \ \Delta G_{0,V}^{\ddagger} - \Delta G_{0,H}^{\ddagger} = -40 \frac{kJ}{mol}\right)$. The equilibrium-potential of the Volmer-step is set to $0.1\,V$. In this case, hydrogen coverage is nearly saturated over the studied potential range. The Tafel slope is $118\frac{mV}{dec}$ and the Constable plot shows a decrease of the apparent activation energy as a function of (negative) overpotential. In Figure S6, we show the Tafel slopes obtained from Figure S2b as a function of overpotential.

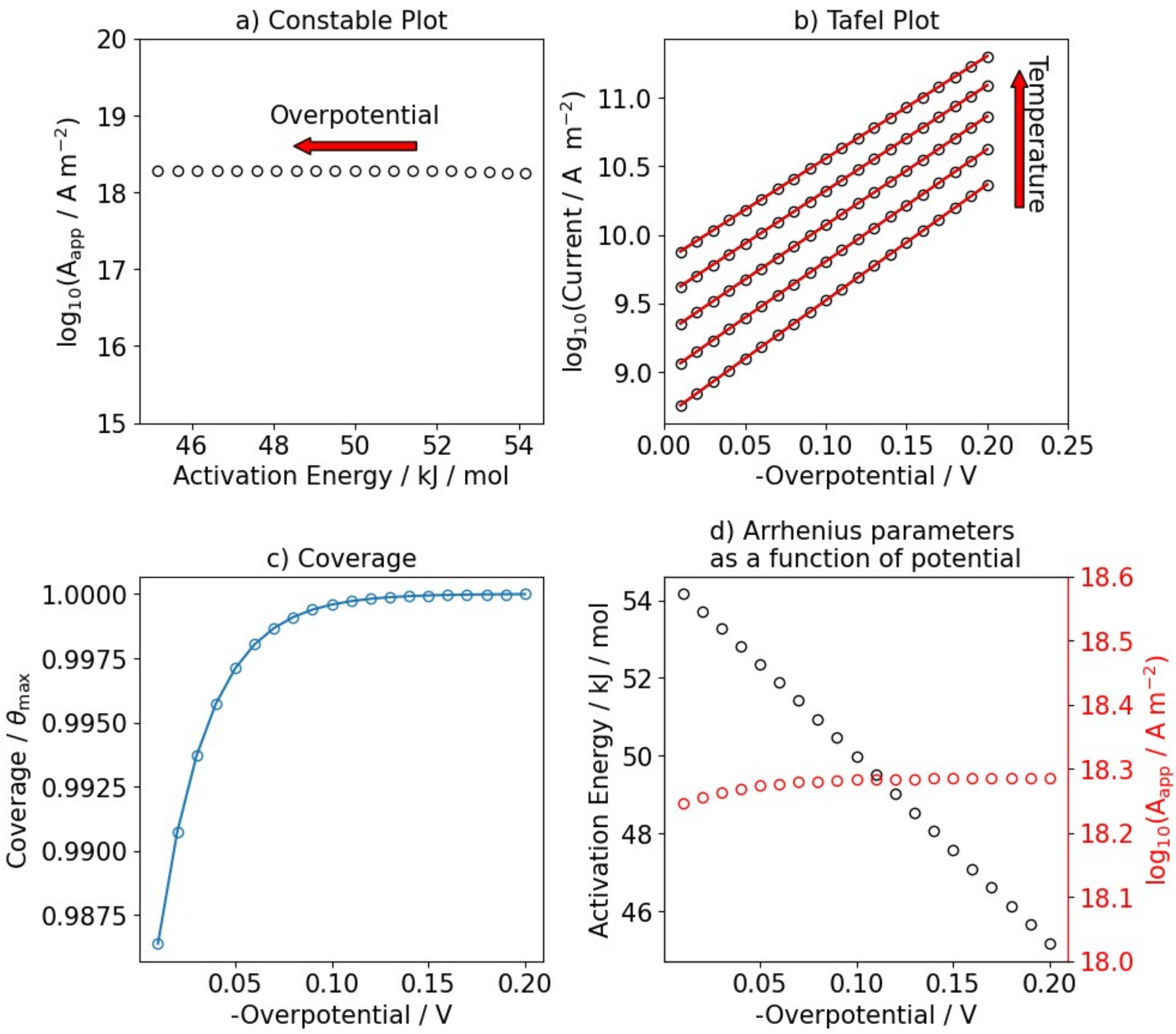

Figure S2: Numerical results of the steady state current of the HER assuming the Volmer-Heyrovsky mechanism. The Heyrovsky step is rate-determining by setting $\Delta G^{\ddagger}_{act,V} \ll \Delta G^{\ddagger}_{act,H}$ namely $\Delta G^{\ddagger}_{act,V}$ = $10\frac{kJ}{mol}$ and $\Delta G^{\ddagger}_{act,H}$ = $50\frac{kJ}{mol}$. The equilibrium potential of the Volmer step is set to $E_V^0 = 0.1\ V$. Panel a) shows the resulting Constable plot. The standard activation energy of the Heyrovsky step is set to $50\ \frac{kJ}{mol}$. The arrow indicates the change of the apparent Arrhenius parameter as a function of applied potential. Panel b) shows the corresponding Tafel plot. The Tafel slope at $298\ K$ is 118 mV/dec. Panel c) shows the steady state coverage of hydrogen in the investigated potential range at $298\ K$. Panel d) shows the apparent Arrhenius parameters (activation energy (black), pre-exponential factor (red)) as a function of applied potential.

Origin of compensation effect. Figure S3 shows the evolution of Gibbs free energy with overpotential for this case, elucidating the contribution of the individual steps to the overall rates. At an overpotential of $-0.01\,V$ (Figure S3b, black), the Heyrovsky step shows a high activation barrier ($\approx 47\frac{kJ}{mol}$) and the normalized surface coverage of adsorbed hydrogen $\left(\frac{H_{ad}}{H_{ad,max}}\right)$ is close to zero, both impeding the reaction rate. Increasing the overpotential to $-0.10\,V$ (Figure S3b, green), the Heyrovsky step becomes more important as the driving force for the Volmer step is sufficiently large to sustain a surface coverage of hydrogen (H_{ad}) that does not impede the Heyrovsky step, which still shows a high activation barrier ($\approx 40\frac{kJ}{mol}$). At a larger overpotential of $-0.20\,V$ (Figure 4b, blue), the activation barrier of the Heyrovsky step becomes low enough such that the reaction mechanism shifts from a pre-equilibrium between protons and adsorbed hydrogen to a sequential two-step process.

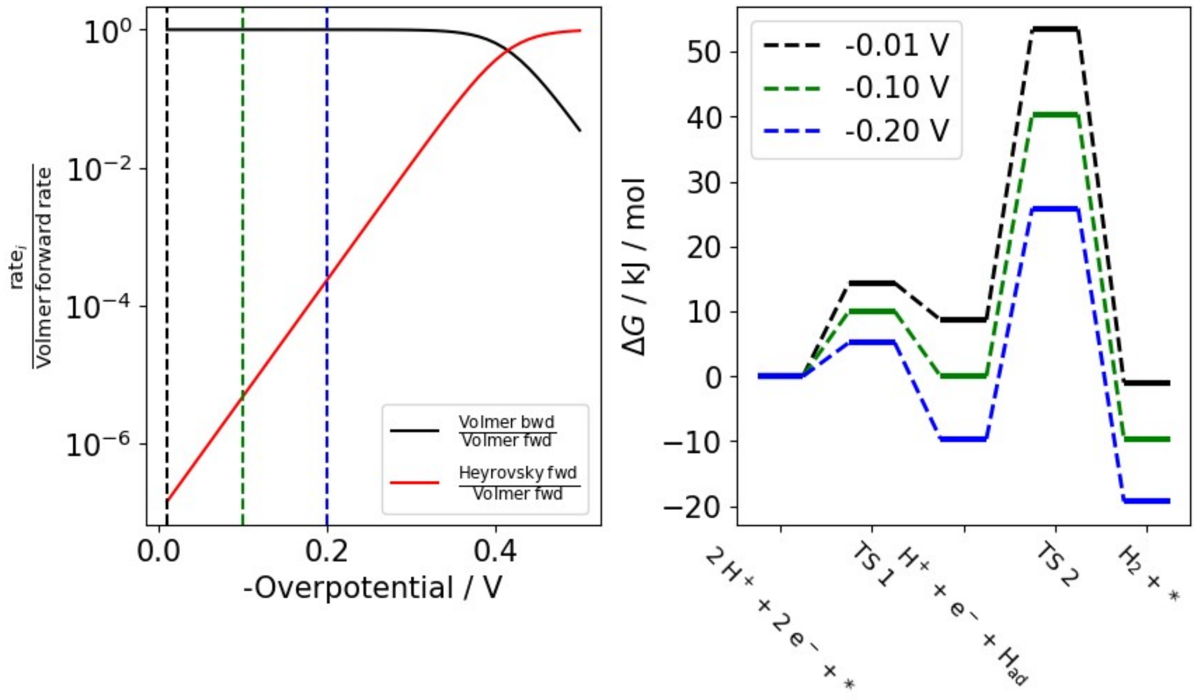

Figure S3: The individual rates that lead to the depopulation of the adsorbed hydrogen state – Volmer backward reaction (second term of Eq. 4) and Heyrovsky forward reaction (third term of Eq. 4) – divided by the Volmer forward reaction over the investigated potential window are shown. Note that the y-axis is logarithmic. While the ratio of the Volmer forward and backward reaction is relatively constant at low overpotentials (black solid line), the change in coverage of hydrogen leads to a change in the contribution of the Heyrovsky forward reaction at higher overpotentials (red solid line).

Volmer-Tafel mechanism. In Figure S4, we show the results of the Volmer-Tafel mechanism when the Volmer-step is set as the RDS. We observe a similar behavior as shown in Figure S1, where the steady-state coverage of hydrogen is essentially zero and the Constable plot shows a linear decrease of the apparent activation energy with applied potential.

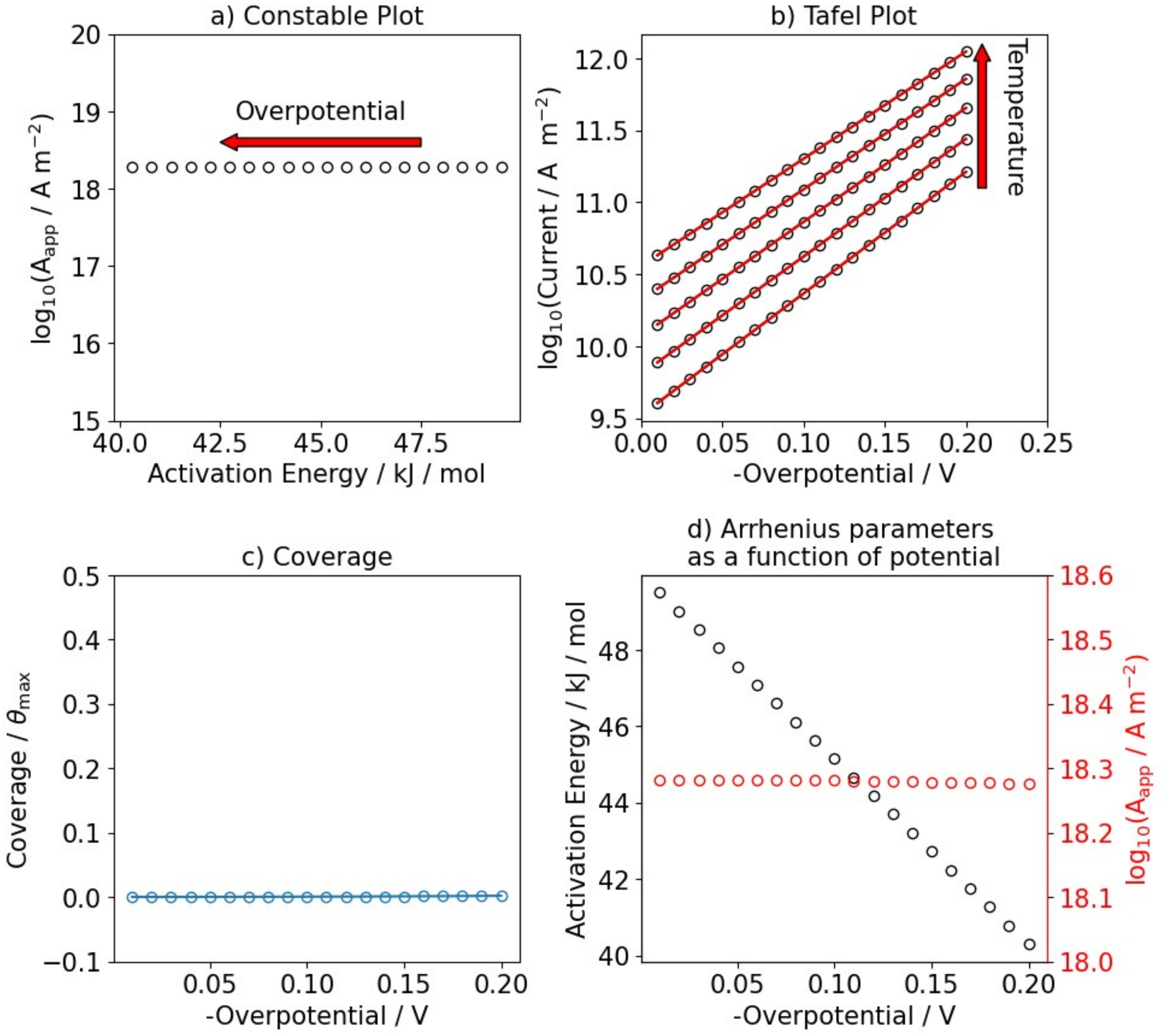

Figure S4: Numerical results of the steady-state current of the HER assuming the Volmer-Tafel mechanism are shown. The Volmer step is assumed to be rate-determining step by setting $\Delta G^{\ddagger}_{act,V} \gg \Delta G^{\ddagger}_{act,T}$, *namely* $\Delta G^{\ddagger}_{act,V}$ = $50\frac{kJ}{mol}$ *and* $\Delta G^{\ddagger}_{act,T}$ = $10\frac{kJ}{mol}$. *The equilibrium potential of the Volmer step is set to* $E^0_V = 0\ V$. *Panel a) shows the resulting Constable plot. The arrow indicates the change of the apparent Arrhenius parameter as a function of applied potential. Panel b) shows the corresponding Tafel plot. The Tafel slope at* $298\ K$ *is 118 mV/dec. Panel c) shows the steady-state coverage of hydrogen in the investigated potential range at* $298\ K$. *Panel d) shows the apparent Arrhenius parameters (activation energy (black), pre-exponential factor (red)) as a function of applied potential.*

Tafel Slopes as a function of overpotential. As proposed by van der Heijden et al.[1-2], we show in Figures S5 to S12 the Tafel slopes as a function of overpotential for all the discussed cases in the main text.

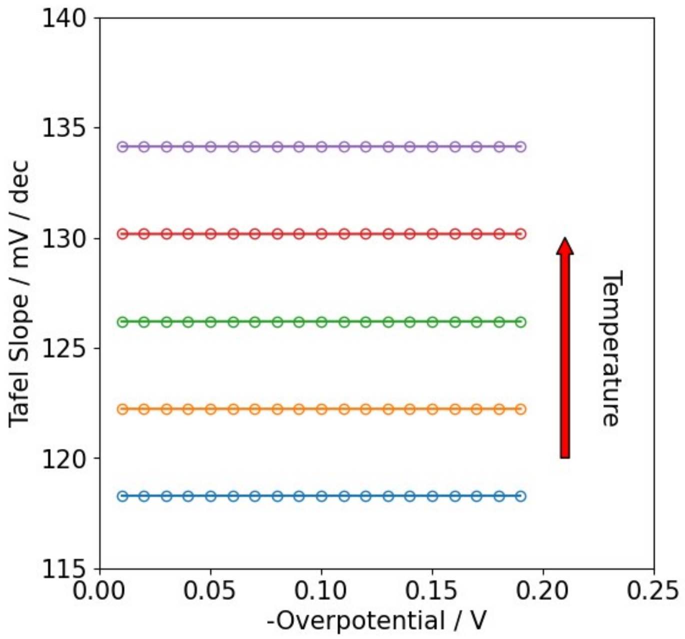

Figure S5: The Tafel slopes of the Volmer-Heyrovsky mechanism when the Volmer step is set as rate determining (see Figure S1) are shown. The Tafel slope increases as a function of temperature as indicated by the arrow.

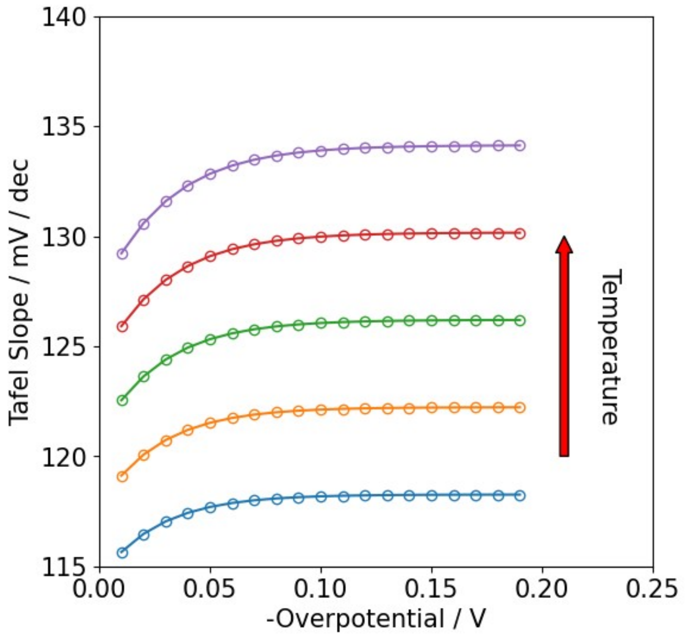

Figure S6: The Tafel slopes of the Volmer-Heyrovsky mechanism when the Heyrovsky step is set as rate determining and the equilibrium potential of the Volmer step is set to $0.1\ V$ *(see Figure 1) are shown. The Tafel slope increases as a function of temperature as indicated by the arrow.*

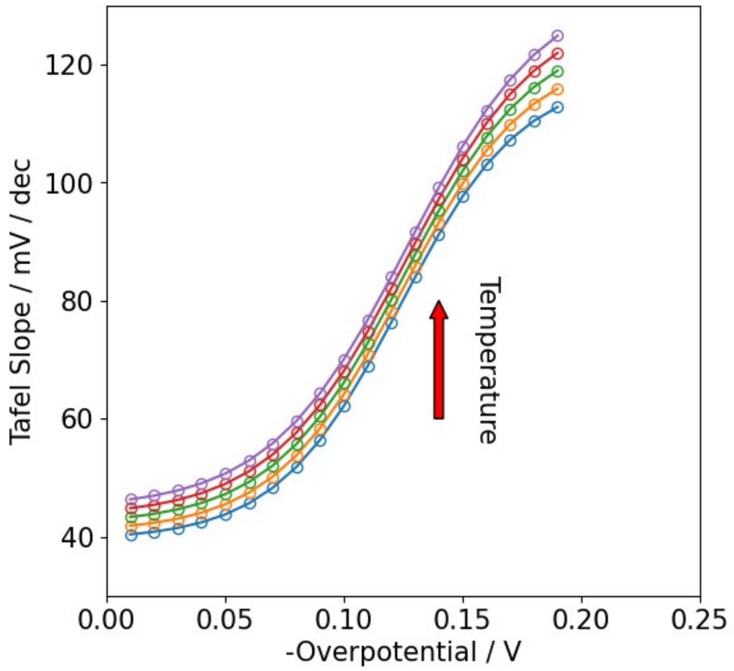

Figure S7: The Tafel slopes of the Volmer-Heyrovsky mechanism when the Heyrovsky-step is set as rate determining and the equilibrium potential of the Volmer step is set to $-0.1\ V$ *(see Figure 2) are shown. The Tafel slope increases as a function of temperature as indicated by the arrow.*

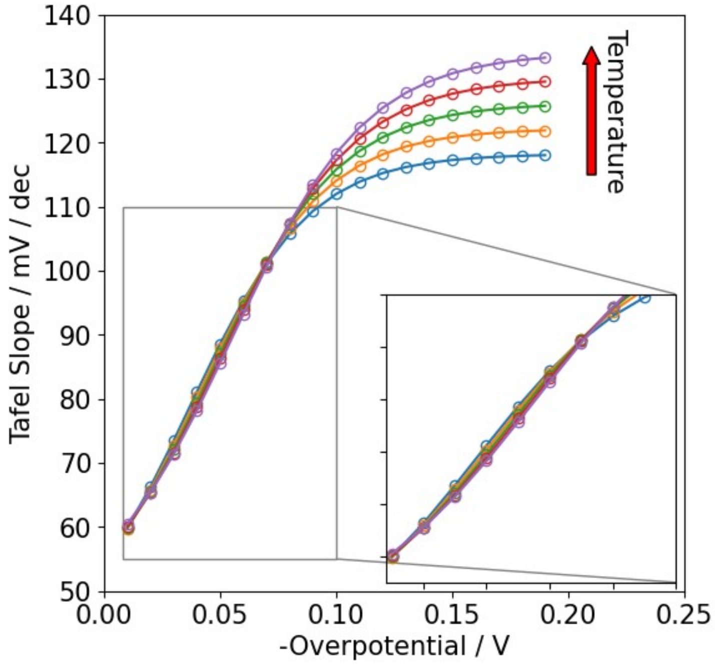

Figure S8: The Tafel slopes of the Volmer-Heyrovsky mechanism when the difference between the activation energy of the Volmer and Heyrovsky step is set to: $\Delta G^{\ddagger}_{act,V} - \Delta G^{\ddagger}_{act,H} = 10\frac{kJ}{mol}$ *and the ratio of the pre-exponential factors to:* $\frac{k_1^0}{k_2^0} = 100$*. The equilibrium potential of the Volmer step is set to* $E_V^0 = -0.1\ V$ *(see Figure 3) are shown. The Tafel slope increases at high overpotentials as a function of temperature as indicated by the arrow.*

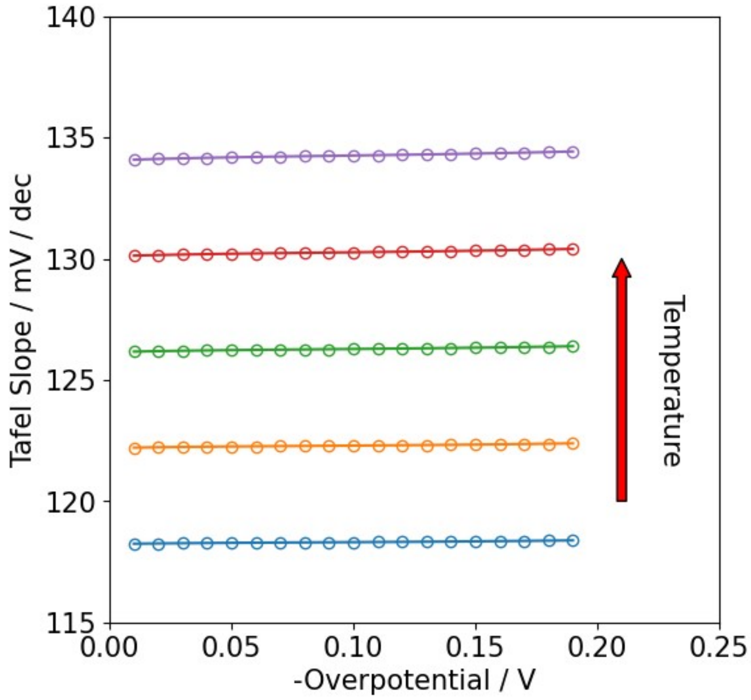

Figure S9: The Tafel slopes of the Volmer-Tafel mechanism when the Volmer-step is set as rate determining (see Figure S4) are shown. The Tafel slope increases as a function of temperature as indicated by the arrow.

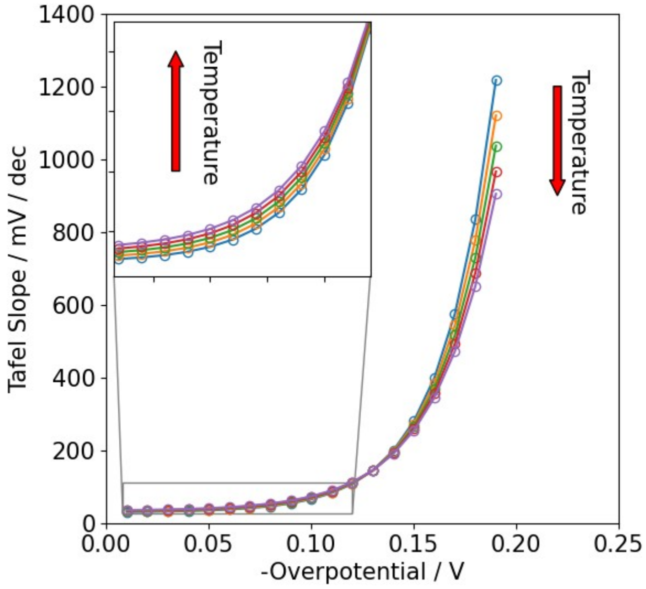

Figure S10: The Tafel slopes of the Volmer-Tafel mechanism when the Tafel-step is set as rate determining (see Figure 5) are shown. The Tafel slope increases as a function of temperature as indicated by the arrow.

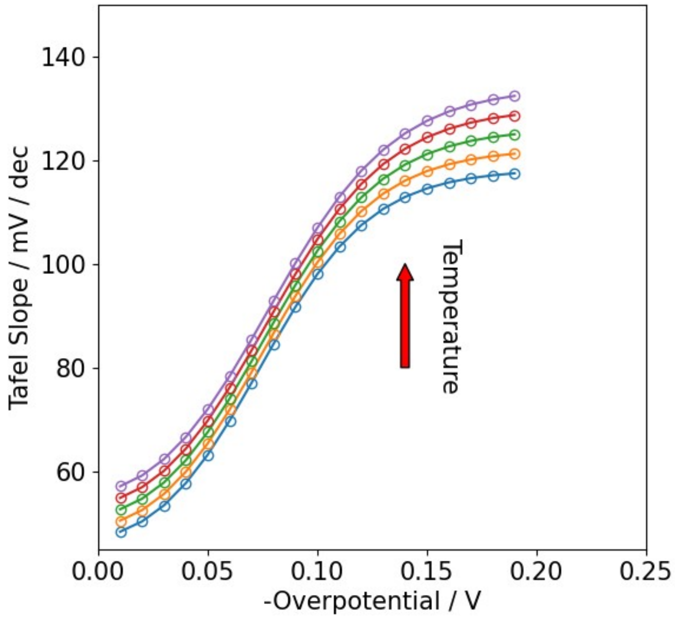

Figure S11: The Tafel slopes of the Volmer-Heyrovsky mechanism when multiple active sites are contributing (see Figure 6). The Heyrovsky step is rate determining by setting $\Delta G^{\ddagger}_{act,V} \ll \Delta G^{\ddagger}_{act,H}$. *The equilibrium potentials of the Volmer step at the two different sites are set to* $E^{0}_{V,1} = 0.15\ V$ *and* $E^{0}_{V,2} = -0.05\ V$ *and the Heyrovsky step is set to be the RDS. The Tafel slope increases as a function of temperature as indicated by the arrow.*

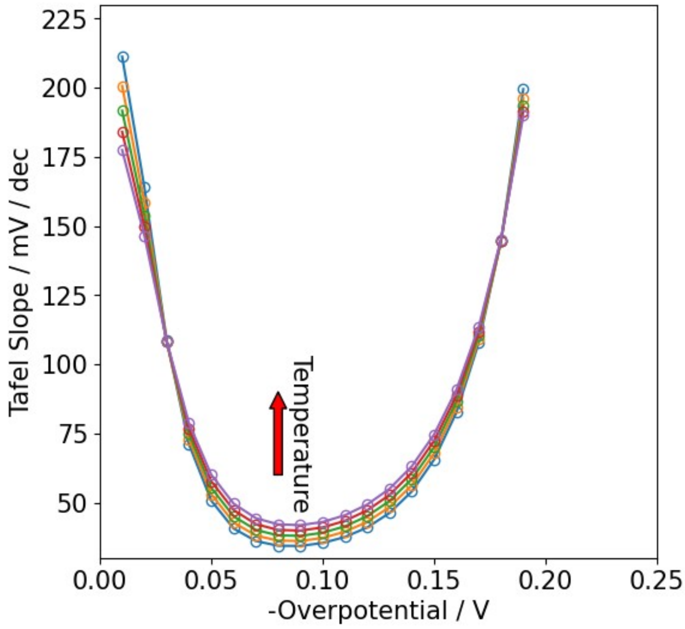

Figure S12: The Tafel slopes of the Volmer-Tafel mechanism when multiple active sites are contributing (see Figure 7). The Tafel step is rate determining by setting $\Delta G^{\ddagger}_{act,V} \ll \Delta G^{\ddagger}_{act,T}$. *The equilibrium potentials of the Volmer step at the two different sites are set to* $E^{0}_{V,1} = -0.15\ V$ *and* $E^{0}_{V,2} = 0.05\ V$.

1. van der Heijden, O.; Park, S.; Vos, R. E.; Eggebeen, J. J. J.; Koper, M. T. M., Tafel Slope Plot as a Tool to Analyze Electrocatalytic Reactions. *ACS Energy Letters* **2024,** *9* (4), 1871-1879.

2. van der Heijden, O.; Vos, R. E.; Koper, M. T. M., Temperature-Dependent Kinetic Parameters for the Alkaline Oxygen Evolution Reaction on NiFeOOH. *ACS Energy Letters* **2025,** *10* (6), 3040-3049.
